# USP14 promotes tryptophan metabolism and immune suppression by stabilizing IDO1 in colorectal cancer

**DOI:** 10.1038/s41467-022-33285-x

**Published:** 2022-09-26

**Authors:** Dongni Shi, Xianqiu Wu, Yunting Jian, Junye Wang, Chengmei Huang, Shuang Mo, Yue Li, Fengtian Li, Chao Zhang, Dongsheng Zhang, Huizhong Zhang, Huilin Huang, Xin Chen, Y. Alan Wang, Chuyong Lin, Guozhen Liu, Libing Song, Wenting Liao

**Affiliations:** 1 Department of Experimental Research, Sun Yat-sen University Cancer Center, State Key Laboratory of Oncology in South China, Collaborative Innovation Center for Cancer Medicine, 510060 Guangzhou, China; 2 Department of Hepatobiliary Surgery, Nanfang Hospital, Southern Medical University, 510515 Guangzhou, China; 3 Department of Pathology, Key Laboratory of Reproduction and Genetics of Guangdong Higher Education Institutes, Key Laboratory for Major Obstetric Diseases of Guangdong Province, The Third Affiliated Hospital of Guangzhou Medical University, 510150 Guangzhou, China; 4 Department of Thoracic Surgery, Sun Yat-Sen University Cancer Center, State Key Laboratory of Oncology in South China, Collaborative Innovation Center for Cancer Medicine, 510060 Guangzhou, China; 5 Department of Pathology, School of Basic Medical Sciences, Southern Medical University, 510515 Guangzhou, China; 6 Department of Biochemistry, Zhongshan School of Medicine, Sun Yat-sen University, 510080 Guangzhou, China; 7 Department of Pathology, Sun Yat-sen University Cancer Center, 510060 Guangzhou, China; 8 Department of Medical Oncology, Sun Yat-sen University Cancer Center, 510060 Guangzhou, China; 9 Key Laboratory of Protein Modification and Degradation, School of Basic Medical Sciences, Guangzhou Institute of Oncology, Tumor Hospital, Guangzhou Medical University, 511436 Guangzhou, China; 10 Brown Center for Immunotherapy, Department of Medicine, Indiana University School of Medicine, Indiana University Melvin and Bren Simon Comprehensive Cancer Center, Indianapolis, IN 46202-3082 USA; 11 School of Life and Health Sciences, The Chinese University of Hong Kong, 518172 Shenzhen, China

**Keywords:** Colon cancer, Cancer microenvironment, Cancer metabolism

## Abstract

Indoleamine 2,3 dioxygenase 1 (IDO1) is an attractive target for cancer immunotherapy. However, IDO1 inhibitors have shown disappointing therapeutic efficacy in clinical trials, mainly because of the activation of the aryl hydrocarbon receptor (AhR). Here, we show a post-transcriptional regulatory mechanism of IDO1 regulated by a proteasome-associated deubiquitinating enzyme, USP14, in colorectal cancer (CRC). Overexpression of USP14 promotes tryptophan metabolism and T-cell dysfunction by stabilizing the IDO1 protein. Knockdown of USP14 or pharmacological targeting of USP14 decreases IDO1 expression, reverses suppression of cytotoxic T cells, and increases responsiveness to anti-PD-1 in a MC38 syngeneic mouse model. Importantly, suppression of USP14 has no effects on AhR activation induced by the IDO1 inhibitor. These findings highlight a relevant role of USP14 in post-translational regulation of IDO1 and in the suppression of antitumor immunity, suggesting that inhibition of USP14 may represent a promising strategy for CRC immunotherapy.

## Abstract (web summary)

IDO1-mediated tryptophan metabolism plays an important role in creating an immunosuppressive tumour microenvironment. Here, the authors show that deubiquitinase USP14 regulates immune suppression by inducing IDO1 stabilization and suggest USP14 as a potential therapeutic target to improve immunotherapy in colorectal cancer.

## Introduction

Immune checkpoint blockade (ICB) therapy has been applied in an increasing number of solid tumors and has significantly improved clinical outcomes in some malignancies^1,2^. However, ICB therapy has limited efficiency in colorectal cancer (CRC)^3^. Lack of antigenic mutations and loss of tumor antigen expression and presentation contribute directly to primary resistance to ICB therapy^4^. Other intrinsic alterations of tumor cells, including enzymatic activity and metabolic disorders, can prevent immune cell infiltration or function within the tumor microenvironment (TME), resulting in a lack of response to immunotherapy^4^. Therefore, strategies designed to disrupt the pathways controlling these metabolic disorders are critical to broadening the application of cancer immunotherapy.

The depletion of the essential amino acid tryptophan (TRP) and the accumulation of kynurenine (KYN) can have a direct negative effect on effector T-cell function, contributing strongly to peripheral immune tolerance^5^. Indoleamine 2, 3 dioxygenase 1 (IDO1) is an essential enzyme that catalyzes the degradation of TRP and the accumulation of KYN^6^. IDO1-mediated TRP metabolism in the KYN pathway is one of the most widely studied metabolic pathways involved in immune tolerance and tumor cell immune evasion. Increased IDO1 expression helps to establish an immunosuppressive TME by inducing the inactivation of T cells and natural killer (NK) cells and the activation and expansion of regulatory T cells (Tregs), dendritic cells (DCs), and myeloid-derived suppressor cells (MDSCs)^5,7,8^. Additionally, KYN and its derivatives can directly interact with the aryl hydrocarbon receptor (AhR) to mediate the activation of AhR signaling^9^. AhR signaling activation has pleiotropic effects on the regulation of the immune response, including upregulating IDO1 expression in tumor cells, upregulating programmed cell death 1 (PD-1) expression in CD8^+^ T cells, upregulating forkhead box P3 (FOXP3) expression in CD4^+^ T cells, impairing CD8^+^ T cells proliferation, and increasing the proportion of Tregs^10–13^. Therefore, IDO1-mediated tryptophan metabolism assists tumor cells in forming an immunosuppressive TME.

Upregulation of IDO1 expression has been identified in various solid tumors. High levels of IDO1 protein are associated with lower CD3^+^ T-cell infiltration in CRC specimens, the presence of distant metastasis, and patient poor survival^14^. Moreover, IDO1-mediated TRP metabolism represents a critical mechanism of resistance to anti-cytotoxic T-lymphocyte associated protein 4 (CTLA-4) or anti-PD-1 therapy^15^, suggesting that targeting IDO1 might act as a potential adjuvant to current immunotherapy. IDO1 inhibitors have been developed and tested in vitro and in vivo as well as in clinical trials^16,17^. However, IDO1 enzyme inhibitors (e.g., Epacadostat) failed to demonstrate therapeutic benefit in combination with ICB in a phase III trial in melanoma^10,18,19^. The mechanisms underlying the failure of IDO1 inhibitors in clinical trials are largely unknown. The “off-target” effects of IDO1 inhibitors are believed to be major reasons for their clinical failure. Potential off-target effects of IDO1 inhibitors mainly include compensatory activation of AhR signaling and activation of the mechanistic target of rapamycin kinase (mTOR) signaling by TRP mimetics in somatic cells^18,20,21^. Thus, beyond inhibiting the enzymatic activity of IDO1, these compounds might simultaneously trigger a wide range of AhR-mediated effects, including altered immune cell differentiation and polarization and upregulation of IDO1 expression^18,20,22^. These findings suggested that the immunomodulatory effect of IDO1 inhibitors is most likely related to their “off-target” effects rather than IDO1 inhibition. Therefore, a better understanding of IDO1 regulation is important to establish alternative approaches to inhibit the IDO1 pathway and improve the efficacy of immunotherapy.

Although abnormal IDO1 expression has been observed in different tumors, the exact mechanisms of its distinct expression patterns are not completely understood. The expression of IDO1 is known to be potently induced at the transcriptional level by type I and type II interferons (IFNs)^23,24^. Other studies revealed that overexpression of cyclooxygenase-2 (COX2) drives constitutive IDO1 expression in human tumors^25^. Moreover, high levels of inflammatory cytokines, such as interleukin (IL)−1, tumor necrosis factor alpha (TNFα), and lipopolysaccharide (LPS), can induce IDO1 expression in epithelial cells or tumor cells^26–28^. In addition to transcriptional regulation, the expression and activity of IDO1 are also controlled at the post-translational level. For example, the suppressor of cytokine signaling 3 (SOCS3) drives proteasomal degradation of IDO1 in DCs under IL-6-driven proinflammatory conditions^29^. In addition, endogenous nitrogen monoxide (NO) binds to IDO1 and reversibly inhibits its activity^30^. Importantly, Liu et al.^31^ reported that IDO1 protein levels, but not mRNA expression, are higher in CRC tissues than in adjacent noncancerous tissues. These studies suggested that post-translational regulation might underlie IDO1 expression and activity under certain circumstances. However, the mechanisms underlying the post-translational regulation of IDO1 in tumor cells are incompletely understood.

Here, using a standardized immune scoring system^32^, we demonstrate that IDO1 levels correlated significantly with the Immunoscore in clinical CRC samples. In addition, we identify ubiquitin-specific protease-14 (USP14) as a key post-translational modifier that maintains high levels of IDO1 in CRC cells. USP14 deubiquitinates and stabilizes IDO1 to prevent its tripartite motif containing 21 (TRIM21, an E3 ligase)-mediated degradation, thereby promoting TRP metabolism and immune suppression in CRC tumors. USP14 inhibition decreases IDO1 protein levels, reverses the immune tolerance of CRC tumors, and sensitizes CRC tumor cells to anti-PD-1 therapy. Importantly, inhibition of USP14 has no off-target effects on AhR activation, which is shown to be driven by traditional IDO1 inhibitors. Together, these findings highlight a vital role of USP14 in the post-translational regulation of IDO1 and suppression of antitumor immunity, establishing USP14 as an important immune therapeutic target in CRC.

## Results

### A reduced Immunoscore is associated with high levels of IDO1 protein in CRC

**Fig. 1:**
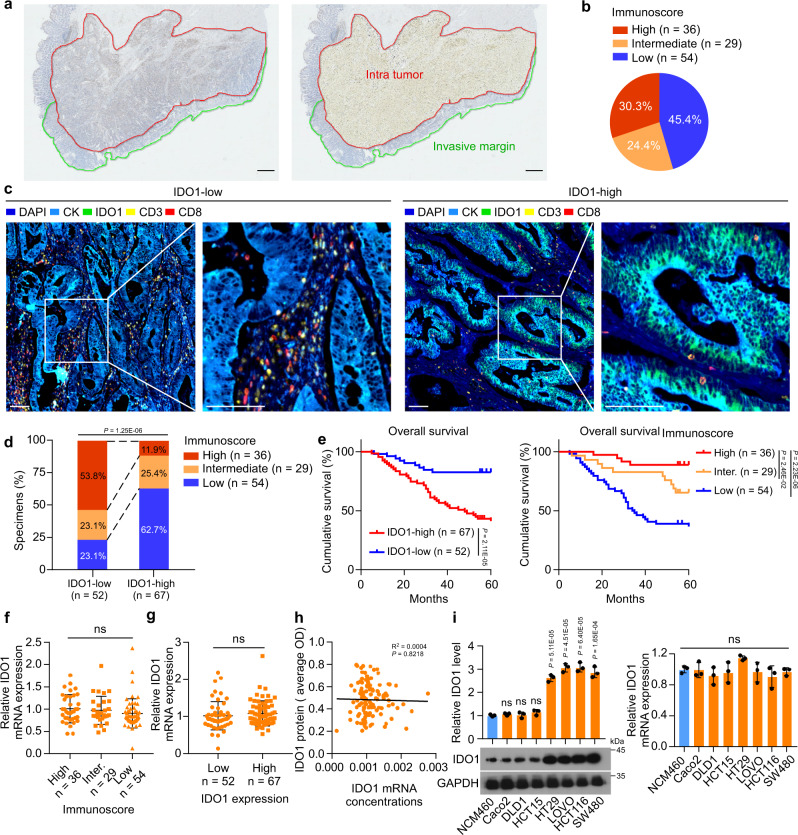
Upregulated IDO1 protein expression is associated with Immunoscore in CRC. **a** Colon tissue was divided into tiles, with tumor tissue highlighted in red and the invasive margin highlighted in green. Scale bars, 1 mm. **b** The percentages of samples with high, intermediate, or low Immunoscore. **c** Representative image showing multiplex immunofluorescence staining of two cases of CRC and the CD3^+^ and CD8^+^ TIL identification strategy. Each marker was represented by a different color, as indicated in the panel. White squares indicated the represent image based in the IDO1, CD3, and CD8 marker expression. Scale bars, 100 µm. **d** Correlation between IDO1 expression and the Immunoscore in 119 human CRC specimens. χ^2^ test (two-sided). **e** Kaplan–Meier analyses of CRC patients stratified by low versus high levels of IDO1 or the Immunoscore (log-rank test, *n* = 119). **f** The relative IDO1 mRNA levels of 119 human CRC specimens related to high, intermediate, or low Immunoscores. **g** The relative IDO1 mRNA levels of 119 human CRC specimens related to low or high levels of IDO1 protein. **h** The correlation between IDO protein levels and IDO1 mRNA expression, according to Spearman correlation analysis (two-sided), R^2^ = 0.004, *P* = 0.8218. **i** Western blotting analysis and quantification of IDO in the indicated cells. The relative mRNA expression of IDO1 in the indicated cells. In **i**, error bars represent the mean ± SD of three independent experiments. ns not significant, two-sided Student’s *t*-test. Source data are provided as a Source Data file.

The Immunoscore measured by CD3^+^/CD8^+^ ^32^ was applied to clarify the clinical relevance of IDO1 protein or mRNA levels in immune cell infiltration in CRC tissues. To calculate the Immunoscore, we performed immunohistochemistry (IHC) to examine CD3 and CD8 levels in 119 CRC samples from patients with stage I–IV colorectal cancer. Halo software was used to determine the mean staining densities of CD3 and CD8 in the tumor center and in the invasive margin, respectively (Fig. 1a, Supplementary Fig. 1a, and Supplementary Table 1). Based on the quantification of CD3^+^ and CD8^+^ T cells, both at the tumor center and at the invasive margin, the samples were stratified into three groups with a low, intermediate, and high Immunoscore (Fig. 1b). Next, IHC and multiplex immunofluorescence analyses were performed to analyze the correlation between IDO1 protein levels and the Immunoscore. The results showed that higher IDO1 protein levels correlated significantly with a lower Immunoscore (Fig. 1c, d, and Supplementary Fig. 1b–d). In addition, patients with a low Immunoscore or high IDO1 protein levels were significantly associated with poor survival (Fig. 1e). However, we found that there was no significant difference in *IDO1* mRNA expression in patients with different Immunoscore or different IDO1 protein levels (Fig. 1f, g). *IDO1* mRNA expression was not associated with the IDO1 protein levels in this cohort of 119 samples (Fig. 1h). In addition, there was no significant difference in *IDO1* mRNA expression in cell lines with different IDO1 protein levels (Fig. 1i). Moreover, IDO1 protein levels, but not mRNA expression, correlated significantly with the production of KYN in CRC samples (Supplementary Fig. 1e–g). These data indicated that IDO1 protein levels, but not mRNA expression, were associated with the infiltration of immune cells in CRC, prompting us to investigate whether IDO1 was under post-translational regulation in CRC.

### USP14 interacts with and stabilizes IDO1 protein in CRC cells

**Fig. 2:**
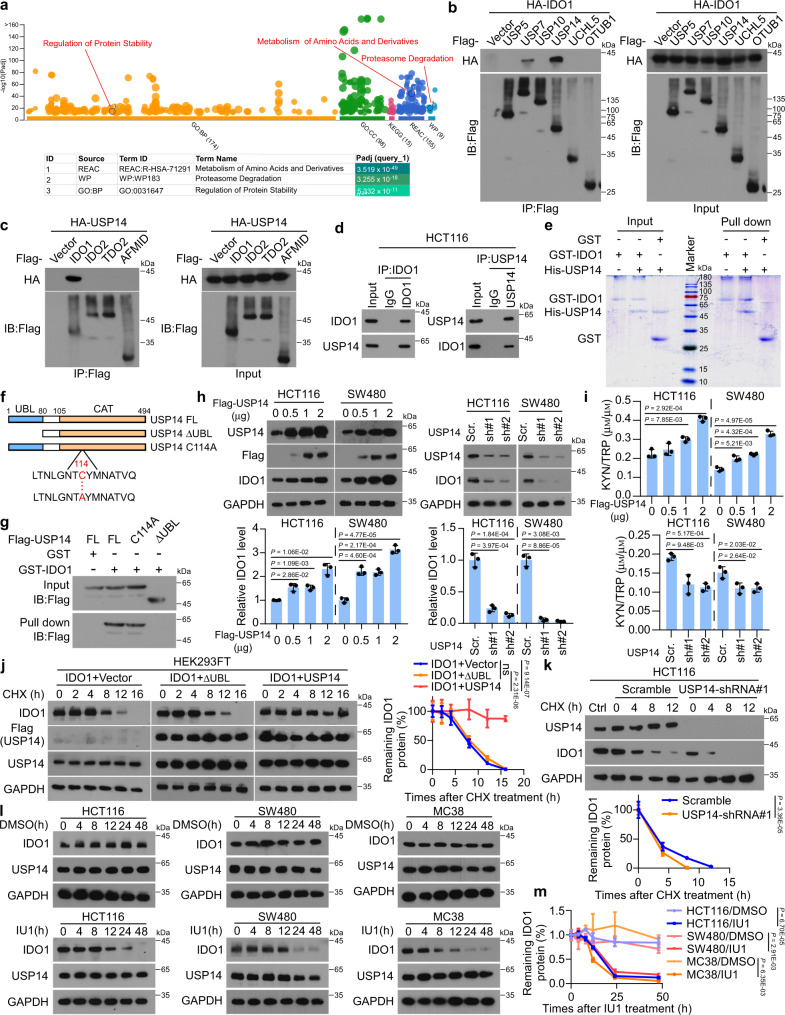
USP14 interacts with and upregulates IDO1 in CRC cells. **a** The results from the MS analysis were analyzed for enrichment. Among the pathways (*P* < 10^6^) with the highest degree of enrichment, two were related to the regulation of protein stability (‘Proteasome Degradation’ and ‘Regulation of Protein Stability’). **b** HEK293FT cells were transfected with Flag-tagged DUBs and HA-IDO1 for 48 h. Lysates were immunoprecipitated with anti-Flag and analyzed. **c** HEK293FT cells were transfected with HA-USP14 and Flag-tagged IDO1, IDO2, TDO2, or AFMID for 48 h. Lysates were immunoprecipitated with anti-Flag and analyzed. **d** HCT116 cells were immunoprecipitated with anti-IDO1 or anti-USP14 and analyzed. **e** Purified USP14 was incubated with GST or GST-IDO1 coupled to GSH-Sepharose, followed by Coomassie blue staining. **f**, **g** HCT116 cells were transfected with the indicated constructs (**f**), and lysates were incubated with GST or GST-IDO1-GSH-Sepharose (**g**). Proteins retained on Sepharose were blotted with the indicated antibody. **h** Western blotting analyses and quantification of the indicated cells transfected with USP14 plasmids, or control shRNA and *USP14* shRNA. **i** KYN and TRP levels were measured using ELISA in the supernatants of cells transfected with USP14 plasmids, or control shRNA and *USP14* shRNA. **j**, **k** HEK293FT cells were transfected with IDO1 and Flag-tagged USP14 or USP14 ΔUBL (**j**) for 48 h, or HCT116 cells were transfected stably with control shRNA and *USP14* shRNA (**k**) were treated with CHX (0.1 mg mL^−1^) for the indicated time, and lysates were analyzed. Graph showing the amount of IDO1 protein remaining after CHX treatment as a percentage of the starting IDO1 protein level. **l** The indicated cells were treated with IU1 (50 µм) or DMSO for the indicated time and lysates were analyzed. **m** Graph showing the amount of IDO1 protein remaining after IU1 treatment as a percentage of the starting IDO1 protein levels. Error bars represent the mean ± SD of three (**h**, **i**) or five (**j**, **k**, and **m**) independent experiments, ns not significant. In **h**, **i**, two-sided Student’s *t*-test. In **j**, **k**, and **m**, One-way repeated-measures ANOVA test. Source data are provided as a Source Data file.

Mass spectrometry (MS) was performed in CRC cells to identify proteins that potentially interact with IDO1. The enriched pathways of IDO1-interacting proteins were analyzed using ingenuity pathway analysis (http://biit.cs.ut.ee/gprofiler/gost), showing that ‘Proteasome Degradation’ and ‘Regulation of Protein Stability’ were among the top enriched pathways related to protein post-translational regulation and degradation (Fig. 2a, and Supplementary Data 1, 2). Several deubiquitinases (DUBs), including USP5, USP7, USP10, USP14, UCHL5, and OTUB1, were found among the proteins in the pathways of proteasome degradation and regulation of protein stability (Supplementary Fig. 2a). The interactions between IDO1 and these DUBs were analyzed using immunoprecipitation (IP), which revealed that only USP14 and USP7 were potent IDO1-interacting proteins (Fig. 2b). Additionally, knockdown of USP14, but not USP7, markedly decreased IDO1 protein levels (Supplementary Fig. 2b). Moreover, overexpression of USP14, but not the other DUBs, increased IDO1 protein levels (Supplementary Fig. 2c). Co-immunoprecipitation (Co-IP) showed that USP14 selectively interacted with IDO1 but not with other enzymes involved in TRP metabolism, including IDO2, tryptophan 2,3 dioxygenase (TDO2), and arylformamidase (AFMID) (Fig. 2c). Moreover, USP14 was upregulated in several CRC cell lines, including HT29, LOVO, HCT116, and SW480 cells (Supplementary Fig. 2d). Immunofluorescence (IF) staining showed strong colocalization of endogenous USP14 and IDO1 in CRC cells (Supplementary Fig. 2e). The interaction between USP14 and IDO1 was confirmed using endogenous reciprocal IP and glutathione-S-transferase (GST) pulldown assays (Fig. 2d, e, and Supplementary Fig. 2f). An active site mutant of USP14 (USP14 C114A) and a truncation mutant of USP14 lacking the ubiquitin-like domain (UBL)^33^ were constructed to determine which domain was responsible for the binding of IDO1 (Fig. 2f). GST pulldown assays showed that the ΔUBL truncation mutant failed to interact with IDO1, indicating that the UBL domain of USP14 was required for the IDO1 interaction (Fig. 2g). These results indicated that USP14 interacted with IDO1 and was involved in the regulation of IDO1 protein levels in CRC.

To evaluate the effects of USP14 on IDO1 protein stability in CRC cells, we examined the expression and half-life of IDO1. The results showed that overexpression of USP14 significantly increased the level of IDO1 in a dose-dependent manner, while knockdown of USP14 using a specific shRNA significantly decreased the level of endogenous IDO1 (Fig. 2h). However, no effects of USP14 on *IDO1* mRNA expression were observed (Supplementary Fig. 2g). Moreover, the KYN/TRP ratio, a potential marker to assess IDO activity, was regulated positively by USP14 (Fig. 2i). Overexpression of wild-type USP14, but not the ΔUBL truncation, prolonged the half-life and increased the level of IDO1 (Fig. 2j). In contrast, knockdown of USP14 shortened the half-life and decreased the level of IDO1 (Fig. 2k). Treatment with IU1, a selective small-molecule inhibitor of USP14^34^, dramatically shortened the half-life and reduced the level of IDO1 (Fig. 2l, m). These results suggested that USP14 interacted with IDO1 and increased the stability of IDO1 protein in CRC cells.

### USP14 deubiquitinates and upregulates IDO1

**Fig. 3:**
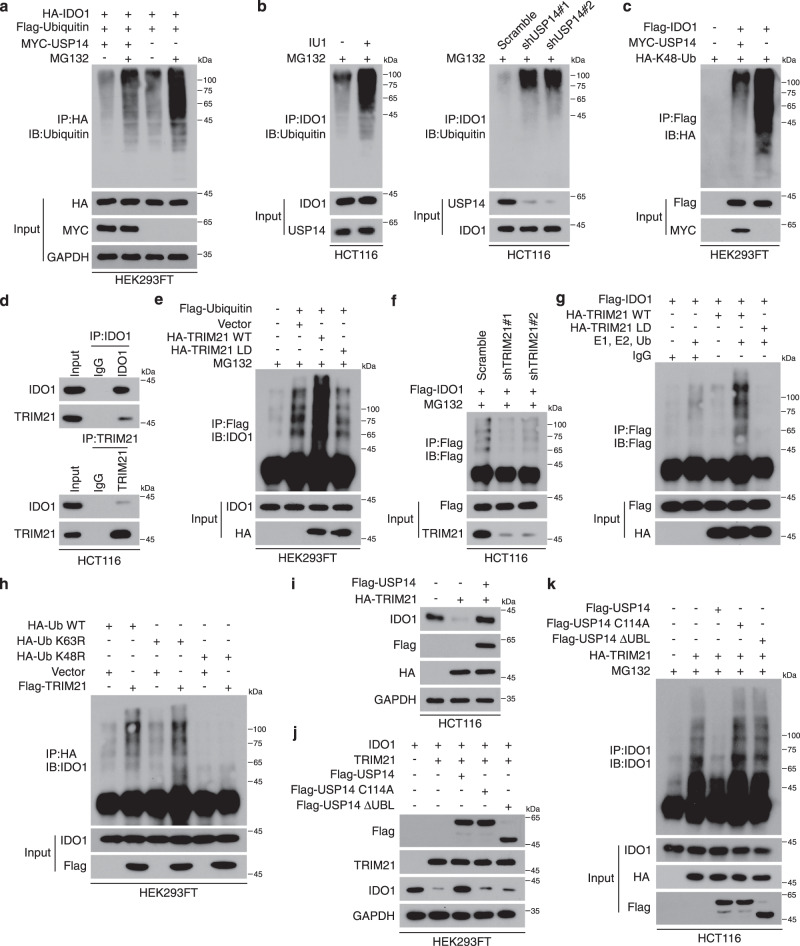
USP14 stabilizes and deubiquitinates IDO1. **a** HEK293FT cells were transfected with HA-IDO1, Flag-Ubiquitin, or MYC-USP14 as indicated. Polyubiquitination of IDO1 was then examined by immunoprecipitation with HA-beads and analyzed. Cells were treated with MG132 (10 µм) for 6 h before being harvested. **b** HCT116 cells treated with IU1 (50 µм) for 24 h or the cells stably transfected with control shRNA and *USP14* shRNA were treated with MG132 (10 µм) for 6 h before being harvested. Lysates were immunoprecipitated with anti-IDO1 and analyzed. **c** HEK293FT cells were transfected with HA-K48-Ub and Flag-IDO1 with or without MYC-USP14 as indicated and cultured for 48 h. Cells were immunoprecipitated with anti-Flag and analyzed. **d** HCT116 cells were immunoprecipitated with anti-IDO1 or anti-TRIM21 and analyzed. **e** Flag-Ubiquitin was transfected together with HA-TRIM21 WT or HA-TRIM21 LD mutant in HEK293FT cells. Cells were treated with MG132 (10 µм) for 6 h before being harvested, then immunoprecipitated with anti-Flag and analyzed. **f** HCT116 cells with stable expression of control shRNA and *TRIM21* shRNA were infected with Flag-IDO1. Cells were treated with MG132 (10 µм) for 6 h before being harvested, then immunoprecipitated with anti-Flag and analyzed. **g** Purified Flag-IDO1 was incubated with IgG control, purified HA-TRIM21 WT, or HA-TRIM21 LD mutant in the ubiquitination assay mix, and the sample was probed for the indicated proteins. **h** HA-Ub (WT, K48R, and K63R mutants) was transfected with or without Flag-TRIM21 in HEK293FT cells. Cells were immunoprecipitated with anti-HA and analyzed. **i** HCT116 cells were transfected with the indicated constructs and analyzed. **j** HEK293FT cells were transfected with IDO1, TRIM21, and Flag-tagged USP14 WT, C114A, or ΔUBL for 48 h and analyzed. **k** HA-TRIM21 was transfected with Flag-USP14 WT, C114A, or ΔUBL in HCT116 cells. Cells were treated with MG132 (10 µм) for 6 h before being harvested, then immunoprecipitated with anti-IDO1 and analyzed. The experiment was repeated three times independently with similar results (**a**–**k**). Source data are provided as a Source Data file.

Proteasomal degradation of IDO1 has been observed in DCs^29^. USP14 significantly increased the stability of IDO1 in CRC cells; therefore, we inferred that USP14 could affect the proteasomal degradation of IDO1. Western blotting analysis showed that IDO1 levels were significantly increased upon treatment with the proteasome inhibitor MG132 in a time-dependent manner (Supplementary Fig. 3a). In addition, cycloheximide (CHX) treatment-induced IDO1 degradation was completely rescued by MG132 treatment, indicating that IDO1 constitutively underwent degradation in a proteasome-dependent manner in CRC cells (Supplementary Fig. 3b). Although IDO1 displayed a notable level of basal ubiquitination, overexpression of USP14 dramatically abolished the endogenous ubiquitination of IDO1 (Supplementary Fig. 3c). Moreover, overexpression of USP14 strongly impaired the ubiquitination of IDO1 (Fig. 3a), while knockdown of USP14 or the addition of IU1 significantly increased IDO1 ubiquitination (Fig. 3b), suggesting that USP14 negatively regulated IDO1 ubiquitination. Next, we investigated whether USP14 regulates IDO1 in a K48 ubiquitination-dependent mechanism. We found that overexpression of USP14 significantly reduced the K48, but not K63, ubiquitination levels of IDO1 (Fig. 3c and Supplementary Fig. 3d). Conversely, both USP14 knockdown, and IU1 addition dramatically increased K48 ubiquitination of endogenous IDO1 (Supplementary Fig. 3e).

As a ubiquitin-specific protease, USP14 deubiquitinated and stabilized the IDO1 protein. Using an in vitro ubiquitination assay, we observed obvious IDO1 ubiquitination in the presence of purified E1 and E2, but without the addition of any E3 ligases (Supplementary Fig. 3f), suggesting that an E3 ligase was immunoprecipitated in the IP samples. These data suggested that E3 ligase(s) controlled the ubiquitination of IDO1. Indeed, the MS analysis of HCT116 and SW480 cells both identified several putative E3 ligases, among which tripartite motifs containing 21 (TRIM21) showed the highest enrichment score. The interaction between IDO1 and TRIM21 was confirmed using co-IP (Fig. 3d). In addition, overexpression of TRIM21 significantly increased the ubiquitination of IDO1 (Fig. 3e). In contrast, TRIM21 knockdown abolished IDO1 ubiquitination upon MG132 treatment (Fig. 3f). An in vitro ubiquitination assay further revealed that TRIM21 directly ubiquitylated IDO1 (Fig. 3g). Moreover, overexpression of the ligase-dead (LD) mutant TRIM21^35^ did not affect the ubiquitination of IDO1, suggesting that the E3 ligase activity of TRIM21 was required for IDO1 ubiquitination (Fig. 3e, g). We further confirmed that TRIM21 ubiquitinated IDO1 protein via the K48-linkage (Fig. 3h). These data indicated that TRIM21 was a direct ubiquitin E3 ligase for IDO1.

We next tested the ability of USP14 to protect IDO1 from TRIM21-mediated degradation. Overexpression of TRIM21 reduced the IDO1 level, while overexpression of USP14 rescued the IDO1 level in the TRIM21-overexpressing cells (Fig. 3i). However, overexpression of USP14 mutants (C114A and ΔUBL) was unable to rescue the level of IDO1 in TRIM21-overexpressing cells (Fig. 3j). Moreover, overexpression of USP14, but not the mutants (C114A and ΔUBL), significantly diminished the ubiquitination of IDO1 in TRIM21-overexpressing cells (Fig. 3k). Co-IP showed that USP14 did not interact with TRIM21 (Supplementary Fig. 3g). These results suggested that USP14 effectively protected IDO1 from TRIM21-induced ubiquitination and degradation.

### USP14 promotes IDO1-mediated immune suppression

**Fig. 4:**
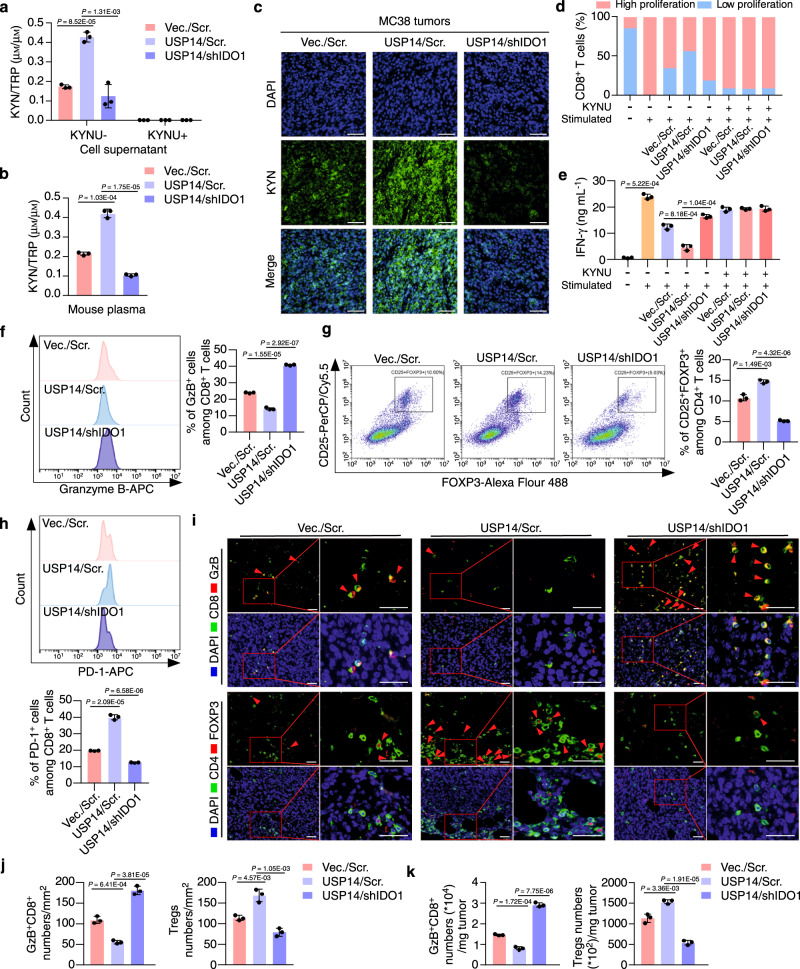
USP14 regulates immune suppression in an IDO1-dependent manner. **a** MC38-Vec./Scr., MC38-USP14/Scr., and MC38-USP14/shIDO1 cells were cultured for 24 h with or without KYNU treatment. KYN and TRP levels in cell supernatants were determined by ELISA (*n* = 3 biological replicates). Vec., vector; Scr., scramble. **b** MC38-Vec./Scr., MC38-USP14/Scr., and MC38-USP14/shIDO1 cells were injected subcutaneously into C57BL/6 J mice at day 0. HPLC-MS/MS analysis was used to generate the tryptophan metabolomic profiles of mouse plasma at day 21, and the KYN/TRP ratio in the mouse plasma was calculated (*n* = 3 biological replicates). **c** Representative immunofluorescence images of the indicated mouse tumors stained with anti-KYN. Scale bars, 100 µm. **d** Quantification of the proliferation of splenic CD8^+^ T cells. Unstimulated T cells were used as a negative control (*n* = 3 biological replicates). **e** IFN-γ secretion by splenic CD8^+^ T cells measured by ELISA (*n* = 3 biological replicates). **f**, **g** The percentage of GzB^+^ cells in CD8^+^ T cells and CD25^+^FOXP3^+^ cells in CD4^+^ T cells isolated from the indicated tumors was measured by flow cytometry and analyzed by FlowJo software. **h** The percentage of PD-1^+^ cells in CD8^+^ T cells isolated from the indicated tumors was measured by flow cytometry and analyzed by FlowJo software. **i**, **j** Representative images and quantification of GzB^+^CD8^+^ cells and FOXP3^+^CD4^+^ cells analyzed by IF staining in tumors (*n* = 3 biological replicates). The small red boxed areas were amplified images of cells. Multiple GzB^+^CD8^+^ cells and FOXP3^+^CD4^+^ cells were marked with red arrowheads. The far-right images in each panel were close-ups of the boxed region. Scale bars, 50 µm. **k** The percentage of GzB^+^CD8^+^ T cells and CD4^+^CD25^+^FOXP3^+^ T cells per milligram of the indicated tumors was measured by flow cytometry and analyzed by FlowJo software (*n* = 3 biological replicates). In **a**, **b**, **e**–**h**, **j**, and **k**, error bars represent the mean ± SD of three independent experiments, two-sided Student’s *t*-test. GzB Granzyme B. Source data are provided as a Source Data file.

IDO1 is under post-translational regulation by USP14; therefore, we speculated that abnormal expression of USP14 could functionally promote TRP metabolism and immune suppression. To address this question, we used MC38 cells stably expressing the *usp14* open reading frame (ORF) or the *usp14* ORF plus *ido1* shRNA (Supplementary Fig. 4a). Overexpression of USP14 significantly increased the KYN/TRP ratio, while knockdown of IDO1 dramatically decreased the KYN/TRP ratio in the supernatants of the USP14-overexpressing cells and in mouse plasma, as assessed using an ELISA or HPLC-MS/MS analysis (Fig. 4a, b, and Supplementary Data 3, 4). The addition of kynureninase (KYNU), an enzyme that degrades kynurenine, abolished the regulation of TRP metabolism by IDO1 or USP14 (Fig. 4a). IF staining showed that overexpression of USP14 markedly increased KYN levels, while knockdown of IDO1 eliminated KYN in the USP14-overexpressing tumors (Fig. 4c).

To examine whether USP14/IDO1-mediated TRP metabolism was linked with T-cell suppression, standard T-cell proliferation co-culture assays were performed (Supplementary Fig. 4b). T-cell suppression assays showed that overexpression of USP14 strongly suppressed anti-CD3/CD28-induced T-cell proliferation and interferon gamma (IFN-γ) secretion, while knockdown of IDO1 abolished the inhibition of T-cell proliferation and IFN-γ secretion in the USP14-overexpressing cells (Fig. 4d, e, and Supplementary Fig. 4c). The addition of KYNU dramatically restored the proliferation and activation of T cells, independent of USP14 and IDO1 expression (Fig. 4d, e, and Supplementary Fig. 4c). To assess the immune suppressive effect of USP14 in vivo, the indicated MC38 cells were subcutaneously injected into C57BL/6 J mice and flow cytometry analyses were performed to examine the infiltration of T cells into tumors. We isolated CD8^+^ T cells and CD4^+^ T cells from the indicated mouse tumors, and the percentage of Granzyme B^+^ or PD-1^+^ cells among CD8^+^ T cells and the percentage of CD25^+^FOXP3^+^ cells among CD4^+^ T cells were detected using flow cytometry. The results showed that overexpression of USP14 dramatically inhibited the percentage of Granzyme B^+^ cells among CD8^+^ T cells while increasing the percentage of CD25^+^FOXP3^+^ cells among CD4^+^ T cells in tumors. However, knockdown of IDO1 restored the percentage of Granzyme B^+^ cells among CD8^+^ T cells while decreasing the percentage of CD25^+^FOXP3^+^ cells among CD4^+^ T cells in the USP14-overexpressing tumors (Fig. 4f, g). In addition, overexpression of USP14 significantly increased the percentage of PD-1^+^ cells among CD8^+^ T cells, while knockdown of IDO1 abolished this increase in USP14-overexpressing tumors (Fig. 4h). These data suggested that overexpression of USP14 impaired the cytotoxic function of CD8^+^ T cells and promoted the differentiation of regulatory T cells. To further detect the infiltration of T cells in tumor tissue, we examined the number of Granzyme B^+^CD8^+^ T cells and FOXP3^+^ Tregs within the tumor tissues. IF staining showed that overexpression of USP14 significantly decreased the infiltration of Granzyme B^+^CD8^+^ T cells and increased the infiltration of FOXP3^+^ Tregs in MC38 tumors, whereas knockdown of IDO1 increased the infiltration of Granzyme B^+^CD8^+^ T cells while decreasing the infiltration of FOXP3^+^ Tregs in the USP14-overexpressing tumors (Fig. 4i, j). These data were confirmed using flow cytometry analyses of the population of Granzyme B^+^CD8^+^ T cells and FOXP3^+^ Tregs per milligram of tumors (Fig. 4k and Supplementary Fig. 4d). Collectively, these data demonstrated that USP14 functioned as a key regulator of immune suppression by modulating IDO1 levels in tumor cells.

### USP14 deficiency reverses immune suppression without activating AhR signaling

**Fig. 5:**
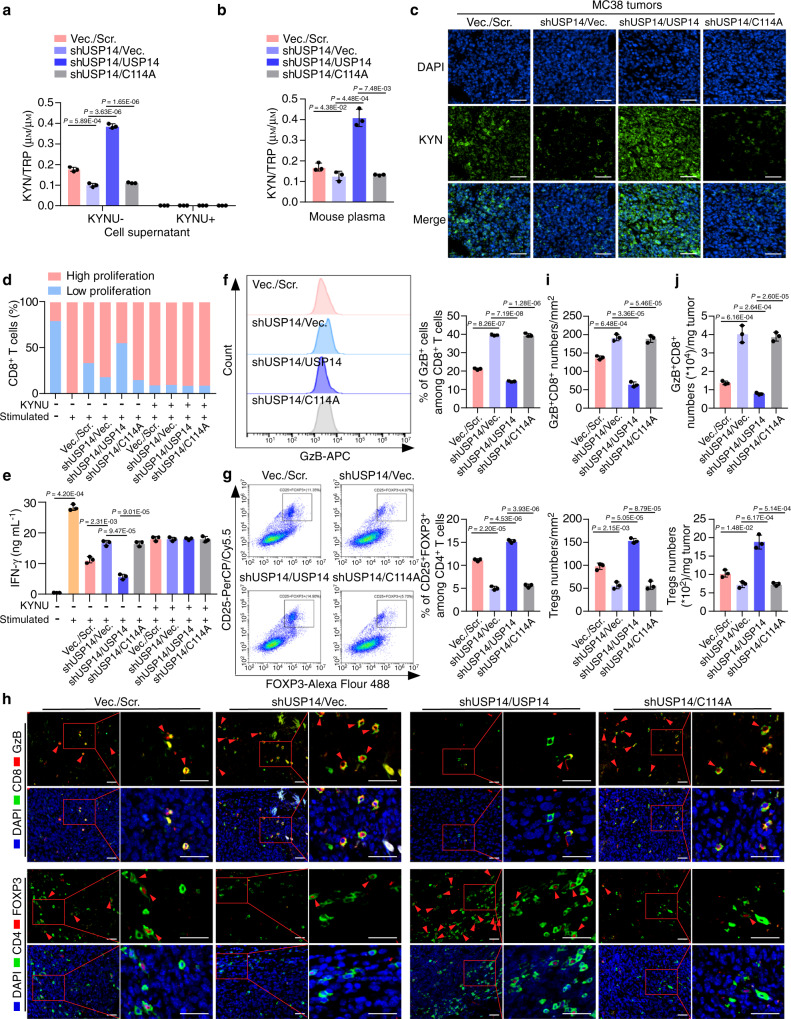
USP14 deficiency reverses immune suppression without activating AhR signaling. **a** MC38-Vec./Scr., MC38-shUSP14/Vec., MC38-shUSP14/USP14, and MC38-shUSP14/C114A cells were cultured for 24 h with or without KYNU treatment. KYN and TRP levels in cell supernatants were determined by ELISA (*n* = 3 biological replicates). **b** MC38-Vec./Scr., MC38-shUSP14/Vec., MC38-shUSP14/USP14, and MC38-shUSP14/C114A cells were injected subcutaneously into C57BL/6 J mice at day 0. HPLC-MS/MS analysis was used to generate the tryptophan metabolomic profiles of mouse plasma at day 21, and the KYN/TRP ratio in the mouse plasma was calculated (*n* = 3 biological replicates). **c** Representative immunofluorescence images of the indicated mouse tumors stained with antibodies against KYN. Scale bars, 100 µm. **d** Quantification of the proliferation of splenic CD8^+^ T cells cultured in the supernatants of the indicated tumor cells. Unstimulated T cells were used as a negative control (*n* = 3 biological replicates). **e** IFN-γ secretion by splenic CD8^+^ T cells cultured in supernatants of the indicated tumor cells, measured by ELISA (*n* = 3 biological replicates). **f**, **g** The percentage of GzB^+^ cells in CD8^+^ T cells (**f**) and CD25^+^FOXP3^+^ cells in CD4^+^ T cells (**g**) isolated from the indicated tumors was measured by flow cytometry and analyzed by FlowJo software. **h**, **i** Representative images and quantification of GzB^+^CD8^+^ cells and FOXP3^+^CD4^+^ cells analyzed by IF staining in tumors (*n* = 3 biological replicates). The small red boxed areas were amplified images of cells. Multiple GzB^+^CD8^+^ cells and FOXP3^+^CD4^+^ cells were marked with red arrowheads. The far-right images in each panel were close-ups of the boxed region. Scale bars, 50 µm. **j** The percentage of GzB^+^CD8^+^ T cells and CD4^+^CD25^+^FOXP3^+^ T cells per milligram of the indicated tumors was measured by flow cytometry and analyzed by FlowJo software (*n* = 3 biological replicates). In **a**, **b**, **e**–**g**, **i**, and **j**, error bars represent the mean ± SD of three independent experiments, two-sided Student’s *t*-test. GzB Granzyme B. Source data are provided as a Source Data file.

To determine whether the enzymatic activity of USP14 was required for its regulation of IDO1-mediated immunosuppression, we silenced endogenous *usp14* using a specific shRNA targeting the 3’UTR. Wild-type USP14 or the enzyme-dead mutant (USP14 C114A) constructs were overexpressed in the endogenous USP14-silenced cells (Supplementary Fig. 5a). The KYN/TRP ratio in the supernatants of the USP14 knockdown cells or mouse plasma was significantly decreased compared with those of the control cells expressing the vector and scrambled shRNA (Fig. 5a, b, and Supplementary Data 5, 6). Overexpression of the wild-type USP14 dramatically increased, while overexpression of the C114A mutant construct failed to change the KYN/TRP ratio in the endogenous USP14-silenced cells or mouse plasma (Fig. 5a, b). The same effects of USP14 or its C114A mutant on the KYN levels were observed in the mouse MC38 tumors (Fig. 5c). In addition, reconstitution of USP14, but not the C114A mutant, suppressed T-cell proliferation and activation, which were abolished by the addition of KYNU (Fig. 5d, e, and Supplementary Fig. 5b). Flow cytometry assays showed that reconstitution of USP14, but not the C114A mutant, decreased the percentage of Granzyme B^+^ cells among CD8^+^ T cells and increased the percentage of CD25^+^FOXP3^+^ cells among CD4^+^ T cells in the USP14-silenced tumors (Fig. 5f, g). Reconstitution of USP14, but not the C114A mutant, significantly increased the percentage of PD-1^+^ cells among CD8^+^ T cells in tumors with silenced endogenous USP14 (Supplementary Fig. 5c). Moreover, reconstitution of USP14, but not the C114A mutant, inhibited the infiltration of Granzyme B^+^CD8^+^ T cells while increasing the infiltration of FOXP3^+^ Tregs in the USP14-silenced tumors (Fig. 5h–j). These data suggested that the DUB activity of USP14 was necessary to promote immunosuppression in CRC.

We next examined whether IU1 would activate AhR signaling^20^. Epacadostat or IU1 were added to the cells. The AhR agonist 2,3,7,8-tetrachlorodibenzo-p-dioxin (TCDD) was used as a positive control. Both Epacadostat and TCDD treatment led to activation of the AhR reporter (Supplementary Fig. 5d), upregulation of IDO1 expression (Supplementary Fig. 5e), and the nuclear translocation of AhR in both tumor cells and splenic T cells (Supplementary Fig. 5f–i). However, IU1 treatment did not cause any changes (Supplementary Fig. 5d–i). AhR activation can enhance the expression of PD-1 in CD8^+^ T cells and FOXP3 expression in CD4^+^ T cells, thus promoting the conversion of CD4^+^ T cells to Tregs and the formation of an immunosuppressive microenvironment. We further tested the effects of IU1 and Epacadostat on CD8^+^ T cells and CD4^+^ T cells isolated from the spleens of MC38 tumor-bearing C57BL/6 J mice. Both Epacadostat and TCDD treatment increased the proportion of PD-1^+^ cells among CD8^+^ T cells and the proportion of CD25^+^FOXP3^+^ cells among CD4^+^ T cells (Supplementary Fig. 5j, k). Interestingly, IU1 treatment did not have any of these AhR activation-mediated effects (Supplementary Fig. 5j, k). Next, we investigated whether we could confirm our experimental findings in vivo. MC38 tumor-bearing mice were treated with KYN, IU1, or Epacadostat. IF staining showed that treatment with KYN and Epacadostat, but not IU1, resulted in nuclear translocation of AhR in mouse tumors (Supplementary Fig. 5l). Moreover, IU1 was more effective in controlling tumor growth in vivo than Epacadostat at equal molar doses in mice bearing MC38 tumors (Supplementary Fig. 5m–o). These data suggested that IU1 might not cause the AhR-mediated off-target effects produced by IDO1 inhibitors.

### Inhibition of USP14 activity increases ICB sensitivity

**Fig. 6:**
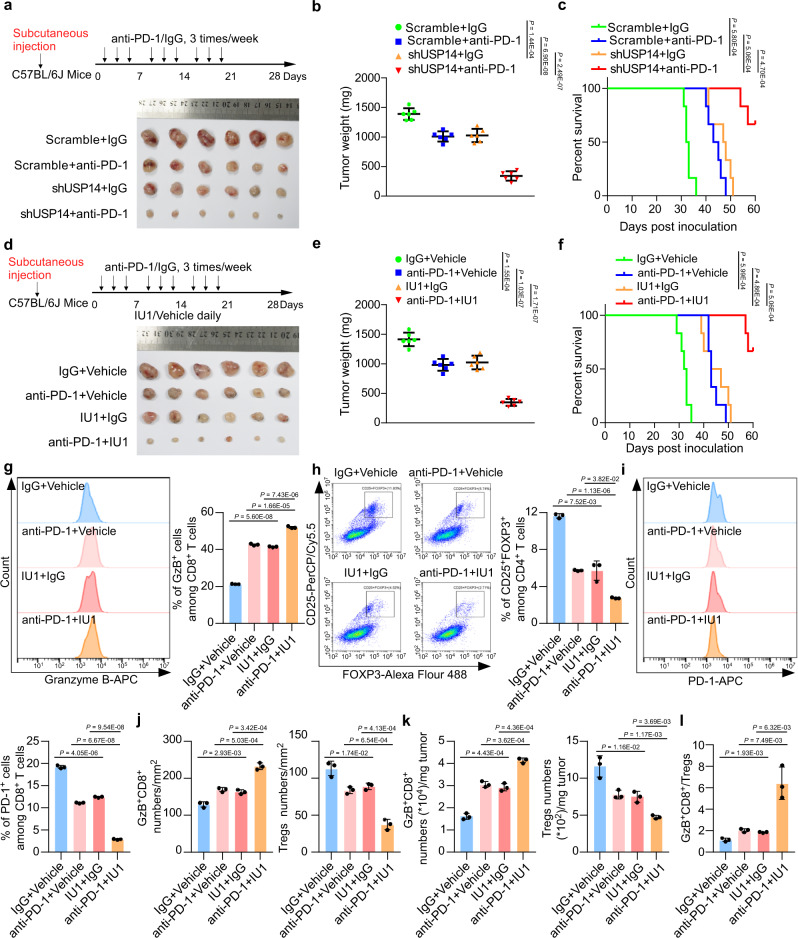
Inhibition of USP14 activity increases ICB sensitivity. **a** Schematic of tumor inoculation and treatment in mice and representative micrographs of tumors. MC38 cells stably transfected with scramble or *usp14* shRNAs were subcutaneously implanted into C57BL/6 J mice at day 0. *n* = 6 for each group. **b** Tumor weight of MC38 cells stably transfected with control shRNA or *usp14* shRNA in C57BL/6 J mice treated with anti-PD-1 or IgG isotype control. *n* = 6 for each group. **c** Survival of the indicated tumor-bearing C57BL/6 J mice treated with anti-PD-1 or IgG isotype control. Log-rank test. *n* = 6 for each group. **d** Schematic of tumor inoculation and treatment in mice and representative micrographs of tumors. MC38 cells were implanted into C57BL/6 J mice subcutaneously at day 0. *n* = 6 for each group. **e** Tumor weight of MC38 cells in C57BL/6 J mice treated with anti-PD-1, IU1, anti-PD-1 plus IU1, or isotype control. *n* = 6 for each group. **f** Survival of MC38 tumor-bearing C57BL/6 J mice treated with anti-PD-1, IU1, anti-PD-1 plus IU1, or isotype control. Log-rank test. *n* = 6 for each group. **g**, **h** The percentage of GzB^+^ cells in CD8^+^ T cells (**g**) and CD25^+^FOXP3^+^ cells in CD4^+^ T cells (**h**) isolated from the indicated tumors was measured by flow cytometry and analyzed by FlowJo software. **i** The percentage of PD-1^+^ cells in CD8^+^ T cells isolated from the indicated tumors was measured by flow cytometry and analyzed by FlowJo software. **j** Quantification of GzB^+^CD8^+^ cells and FOXP3^+^CD4^+^ cells analyzed by IF staining in tumors (*n* = 3 biological replicates). **k** The percentage of GzB^+^CD8^+^ T cells and CD4^+^CD25^+^FOXP3^+^ T cells per milligram of the indicated tumors was measured by flow cytometry and analyzed by FlowJo software (*n* = 3 biological replicates). **l** Ratio of GzB^+^CD8^+^ T cells to Tregs in the indicated tumors. In **g**–**l** error bars represent the mean ± SD of three independent experiments. In **b**, **e**, and **g**–**l**, two-sided Student’s *t*-test. GzB Granzyme B. Source data are provided as a Source Data file.

Next, we explored the effect of USP14 knockdown or inhibition of USP14 activity on the efficacy of anti-PD-1 therapy using the MC38 syngeneic mouse model. The mice bearing *usp14* shRNA-expressing MC38 cells showed moderately decreased tumor weights and increased survival compared with the mice implanted with control MC38 cells (Fig. 6a–c and Supplementary Fig. 6a). More importantly, intraperitoneal administration of anti-PD-1 exerted more effective tumor suppression and survival improvement in the *usp14* shRNA group than in the control group (Fig. 6a–c and Supplementary Fig. 6a). In addition, IU1 as a single agent, slightly reduced the mouse tumor burden and improved survival rates, while the combined IU1 and anti-PD-1 treatment significantly reduced tumor weights and extended the survival of the mice (Fig. 6d–f and Supplementary Fig. 6b, c). Flow cytometry analyses showed that IU1 or anti-PD-1, as single agents slightly, while the combination of IU1 and anti-PD-1 significantly, increased the proportion of Granzyme B^+^ cells among CD8^+^ T cells, decreased the proportion of CD25^+^FOXP3^+^ cells among CD4^+^ T cells, and decreased the proportion of PD-1^+^ cells among CD8^+^ T cells in the tumors (Fig. 6g–i). The effects of IU1, anti-PD-1, or both on the infiltration of Granzyme B^+^CD8^+^ T cells and FOXP3^+^ Tregs were confirmed by IF staining and flow cytometry (Fig. 6j, k, and Supplementary Fig. 6d). Moreover, the combination of anti-PD-1 and IU1 effectively increased the Granzyme B^+^CD8^+^/Tregs ratio in the tumor mass, which is characteristic of successful ICB-based therapy^36^ (Fig. 6l). Furthermore, the number of cleaved caspase-3-positive tumor cells increased significantly in the combination treatment group, indicating that inhibition of USP14 together with anti-PD-1 was a highly effective therapeutic strategy for CRC (Supplementary Fig. 6e). These results demonstrated that inhibition of USP14 activity significantly increased the sensitivity of MC38 tumors to anti-PD-1 therapy.

### Clinical relevance of USP14 and IDO1 in immune infiltration

**Fig. 7:**
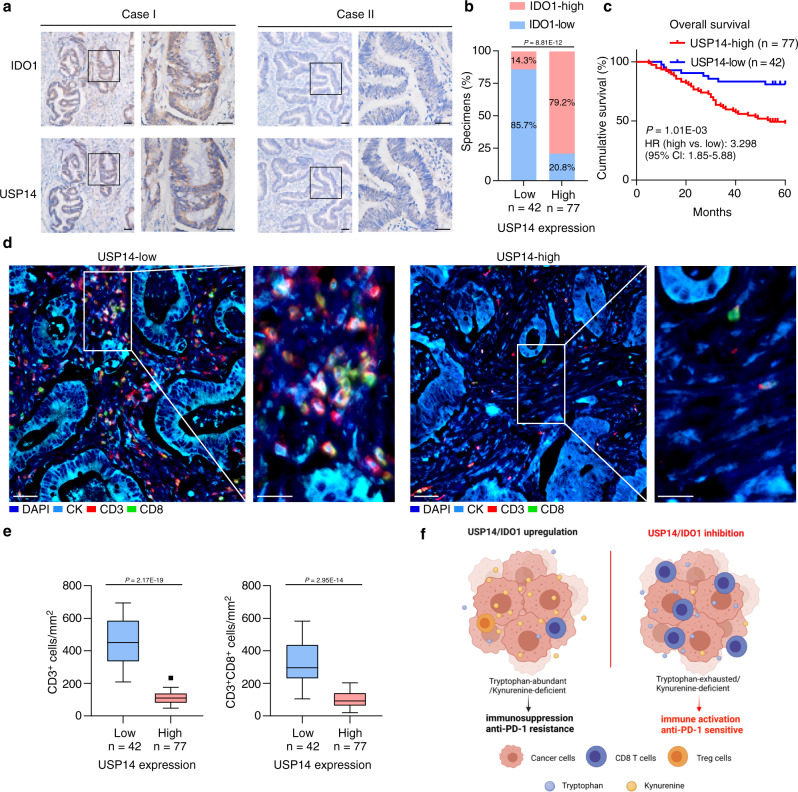
Clinical relevance of USP14 in CRC patients. **a** Representative images of USP14 and IDO1 IHC staining in human CRC specimens. Scale bars, 50 µm. **b** Percentage of samples showing low or high IDO1 levels in 119 human CRC specimens relative to the level of USP14. χ^2^ test (two-sided). **c** Kaplan–Meier analysis for CRC patients stratified by high versus low levels of USP14 (log-rank test, *P* = 0.001, *n* = 119). **d** Representative images showed multiplex immunofluorescence staining of two cases of CRC and the tumor infiltrating lymphocyte (TIL) identification strategy. Each marker was represented by a different color as indicated in the panel, scale bars, 50 µm. White boxes indicated the representative image, based in the CD3 and CD8 marker expression. CK cytokeratin. Scale bars, 25 µm. **e** Quantification of CD3^+^ and CD3^+^CD8^+^ T cells analyzed by multiplex immunofluorescence staining in USP14-high (*n* = 77) and USP14-low (*n* = 42) CRC tissues. The lines represented the minimum, first quartile, median, third quartile, and maximum of the number of CD3^+^ and CD3^+^CD8^+^ T cells. Two-sided Student’s *t*-test. **f** Schematic representations of the role of the USP14/IDO1 axis in immune suppression and ICB resistance in CRC (Created with BioRender.com). Source data are provided as a Source Data file.

To assess the correlation between USP14 and IDO1 levels in clinical CRC samples, we performed IHC in 42 specimens with low levels of USP14 and 77 specimens with high levels of USP14 (Fig. 7a). A total of 79.2% of the USP14-high samples showed high IDO1 levels compared with 14.3% of the USP14-low samples (Fig. 7b). High USP14 levels correlated significantly with N classification (*P* = 0.047; χ^2^ test) and IDO1 levels (*P* < 0.001; χ^2^ test) (Supplementary Table 2). Importantly, Kaplan–Meier survival curves and log-rank tests revealed that patients with high USP14 expression had a shorter 5-year overall survival (*P* = 0.001; hazard ratio = 3.298 (95% confidence interval (CI) 1.85–5.88)) (Fig. 7c). In addition, multivariate Cox regression analyses indicated that USP14 levels and N and M classification were independent prognostic factors for 5-year overall survival in CRC (Supplementary Table 3). To evaluate the correlation between USP14 and antitumor immunity in CRC, we performed multiplex immunofluorescence analysis using anti-keratin, anti-CD3, and anti-CD8 antibodies in CRC samples. Samples with high USP14 levels showed dramatically decreased infiltration of CD3^+^ cells and CD3^+^CD8^+^ T cells (Fig. 7d, e). The multiplex immunofluorescence analysis of USP14, IDO1, and CD8 in two CRC specimens also showed that the high levels of USP14 and IDO1 were negatively associated with the infiltration of CD8^+^ T cells (Supplementary Fig. 7a). These results showed that the USP14/IDO1 axis correlated negatively with antitumor immune infiltration in CRC tumors, further supporting the view that inhibition of USP14 could enhance CRC sensitivity to anti-PD-1 therapy (Fig. 7f).

## Discussion

In this study, we identify USP14 as an important post-translational regulator of IDO1 that maintains high levels of IDO1 in CRC. Overexpression of USP14 in CRC cells deubiquitinates and stabilizes IDO1 to protect against TRIM21-mediated ubiquitination and degradation via proteosomes, thereby promoting TRP metabolism and suppressing T-cell proliferation and activity in CRC. In addition, knockdown of USP14 or inhibition of USP14 activity decreases IDO1 protein levels, suppresses TRP metabolism, and impairs CD8^+^ T cells proliferation and activation and the conversion of CD4^+^ cells to Tregs. Moreover, knockdown of USP14 or inhibition of USP14 activity increases the antitumor immune response and the efficacy of anti-PD-1 therapy in CRC tumors. Finally, we establish a link between USP14 and the suppression of antitumor immunity in clinical CRC samples. Our study reveals the molecular mechanism of IDO1 post-translational modification, which might represent a feasible tumor-targeting strategy and an alternative immunotherapy method to directly inhibiting IDO1.

IDO1 is frequently overexpressed in cancer cells and stromal cells in the TME^37^. IDO1 expression is widely stimulated at the transcriptional level by inflammatory molecules such as IFNs, TNF-α, pathogen-associated molecular patterns (PAMPs), and damage-associated molecular patterns (DAMPs). In addition, *IDO1* mRNA expression is upregulated by oncogenic signaling pathways, including KIT proto-oncogene, receptor tyrosine kinase (KIT), KRAS proto-oncogene, GTPase (RAS), Janus kinase (JAK)-signal transducer and activator of transcription (STAT), and nuclear factor kappa B (NF-κB) pathways^38–40^. Although numerous works have revealed various transcriptional mechanisms that induce IDO1 expression in tumors, recent studies have demonstrated that post-translational mechanisms also affect the expression and activity of IDO1 under certain circumstances^41,42^. IDO1 harbors immunoreceptor tyrosine-based inhibitory motifs (ITIM1 and ITIM2), which act as docking sites for different molecular partners and activate the post-translational modulation of IDO1^43^. The half-life of IDO1 is controlled by the ubiquitin-proteasome system; however, the specific post-translational regulatory mechanism of IDO1 remains largely unclear. In the current study, we demonstrate that upregulated IDO1 levels are associated with a reduced Immunoscore in CRC, and there is no correlation between IDO1 protein and mRNA levels, implying that the abundance and activity of IDO1 are more likely under post-translational regulations in CRC. In addition, we identify USP14, a DUB reversibly associated with the proteasome^34^, as an IDO1-interacting partner using mass spectrometry analysis. Moreover, USP14 directly deubiquitinates and stabilizes IDO1 to protect it against ubiquitination and proteosome-mediated degradation in cancer cells. USP14 has been shown to negatively regulate proteasome activity by trimming K48 ubiquitin chains on proteasome-bound substrates. For example, USP14 cleaves the K48-linked ubiquitination of cyclic GMP-AMP synthase (cGAS) and inhibits cGAS degradation^44^. TRIM21 is an E3 ligase that induces K48 ubiquitination and has been reported to promote the degradation of DEAD-box helicase 41 (DDX41) in DCs^45^. Herein, we identify TRIM21 as a key E3 ligase that promotes K48 ubiquitination and degradation of IDO1, while USP14 inhibits TRIM21-mediated K48 ubiquitination of IDO1, thereby enhancing the abundance of IDO1. Thus, these findings identify a post-translational mechanism responsible for the high abundance and activity of IDO1 in CRC cells.

E3 ubiquitin ligases and deubiquitinating enzymes (DUBs) play essential roles in protein homeostasis through ubiquitination and deubiquitination. As a DUB that is reversibly associated with the 19 S proteasome, USP14 protects proteins from degradation by cleaving the ubiquitin chain from its substrate distal tip. It has been reported that USP14 mediates the deubiquitination of Dishevelled (Dvl)^46^ and NLR family CARD domain containing 5 (NLRC5)^47^ to regulate the Wnt and NF-κB pathways. In addition, USP14 directly interacts with and increases the stability of fatty acid synthase (FASN) to promote hepatosteatosis^48^. Moreover, USP14 is involved in the suppression of the antiviral immune response by promoting K63‐linked retinoic acid-inducible gene 1 protein (RIG‐I) deubiquitination, and IU1 enhances RIG-I-triggered IL-6 and TNF-α expression in vesicular stomatitis virus (VSV)-infected mice in vivo. These results demonstrate that USP14 is a potential target for RNA virus-related diseases^49,50^. USP14 activation promotes tumor progression by increasing cell proliferation and inhibiting apoptosis^51,52^. Importantly, there are a few studies linking USP14 to immune suppression. For example, USP14 also promotes cytokine release and autophagy by regulating canonical and noncanonical NF-κB signaling^53,54^. In addition, USP14 deubiquitinates and increases the stability of C-X-C Motif chemokine receptor 4 (CXCR4), which is highly expressed in tumors and plays an important role in tumor immune suppression^55,56^. USP14 is also involved in major histocompatibility complex I (MHC I) antigen presentation by regulating the ubiquitination of peptides^57^. These studies identify a potential oncogenic role of USP14 in tumorigenesis and immune regulation. However, the direct substrates and additional roles of USP14 remain largely unknown. In the current study, we demonstrate a critical role of USP14 in TRP metabolism and immune suppression. Our data show that IDO1 is a proteolysis-associated substrate that interacts directly with USP14. Overexpression of USP14 effectively deubiquitinates and stabilizes IDO1 in CRC cells, thereby promoting TRP metabolism and suppressing antitumor immunity. This notion is confirmed using the MC38 syngeneic mouse model, which shows that both USP14 knockdown and an inhibitor restrain tumor growth, increase the infiltration of cytotoxic T cells, decrease the infiltration of Tregs, and finally promote the response to anti-PD-1 therapy. The small-molecule inhibitor IU1 specifically and selectively inhibits USP14 by disrupting its ubiquitin chain-shortening activity^34^. Previous studies have demonstrated that IU1 exerts an antitumor role by reducing cell growth and triggering cell apoptosis in cervical cancer and breast cancer^58–60^. In the current study, we demonstrate that IU1 dramatically reduces IDO1 protein levels and suppresses IDO1-mediated TRP metabolism and immune suppression while abolishing the “off-target” effects of IDO1 inhibitors on AhR activation. Therefore, our results from the preclinical animal model suggest that IU1 also attenuates immune suppression and improves the efficacy of immunotherapy. Furthermore, our results demonstrate that the UBL domain of USP14 is necessary for the interaction between USP14 and IDO1. Thus, inhibitors targeting the UBL domain would specifically abolish the interaction between USP14 and IDO1. Unfortunately, these inhibitors are currently not available. In the present study, we find that IU1, which targets the catalytic site of USP14^34^, could effectively downregulate IDO1 (Fig. 2l). In addition, overexpression of the enzyme-dead mutant (USP14 C114A) fails to rescue IDO1 levels (Supplementary Fig. 5a). Therefore, IU1 might have a similar effect as inhibitors specifically targeting the UBL domain to regulate IDO1 expression and IDO1-related immune suppression. Nevertheless, it is worth developing inhibitors that specifically target the UBL domain.

There is still an unmet clinical need for immunotherapy for most patients with CRC who do not respond to ICB. IDO1 is an important innate immune regulator that acts by triggering TRP metabolism in the TME. Targeting IDO1 represents an opportunity in cancer immunotherapy beyond ICB. However, IDO1 inhibitors have suffered major setbacks in recent clinical trials. The reasons for this negative result are not well defined. Recent studies indicated that TRP-related IDO1 inhibitors might have potential off-target effects. This effect is mainly because these compounds (including 1-MT, Epacadostat, Navoximod, and Norharmane) potently activate AhR-mediated signaling, followed by immune suppression and upregulation of IDO1 expression^20^. AhR can be activated by tryptophan metabolites such as KYN^61^, 6-formylindolcarbazole (FICZ, a photoproduct of TRP)^62^, and KYNA^63^. For instance, AhR is activated by IL4I1 through the generation of indole metabolites Indole-3-propionic acid (I3P) and KYNA^64^. IDO1 inhibitors do not block IL4I1; therefore, IL4I1 activity might partially explain the failure of clinical studies with IDO1 inhibition^64^. In addition, AhR is phosphorylated and activated by protein kinase C (PKC)^65^. 1-methyl-D-tryptophan (D-1MT), a potent TRP mimetic, restores the PKC activity in cells with IDO-mediated TRP deprivation^21^. These data suggest that IDO1 inhibitors might also activate AhR by activating PKC. Nevertheless, AhR activation is not the only factor that is believed to underlie the failure of IDO1 inhibitors in cancer treatment. TRP-related IDO inhibitors mimic TRP as fake nutritional signals might activate mTOR signaling in somatic cells^21^. Other reasons for the lack of effective IDO1 inhibition could be dose-limiting toxicities and drug levels that are too low to inhibit IDO1 activity in vivo^66^. Moreover, the tumor penetration of small-molecule inhibitors is worthy of discussion. It has been reported that IU1 can inhibit the growth of subcutaneous tumors, suggesting the favorable penetration of IU1 into tumor tissues in mice^67^.

In summary, we demonstrate that IU1 dramatically reduces IDO1 protein levels and suppresses IDO1-mediated TRP metabolism and immune suppression while abolishing the off-target effects of IDO1 inhibitors on AhR activation. Therefore, our results from preclinical animal models suggest that suppressing USP14 via IU1 might represent a promising alternative approach to effectively inhibit IDO1-mediated immune suppression and improve the efficacy of immunotherapy. Generally, USP14 is a promising drug target for cancer therapy, especially in patients with high IDO1 expression. Thus, targeting IDO1 stabilization through USP14 inhibition represents a potential immunotherapeutic strategy and combinational strategy with ICB reagents in CRC.

## Methods

### Tumor models and treatments

Female C57BL/6 J mice, 6–8 weeks old, were purchased from the Center of Medical Experimental Animals of the Chinese Academy of Medical Science (Beijing, China) and housed in a barrier facility on a 12 h light/dark cycle at 18–22 °C and 50–60% humidity. The Institutional Animal Care and Use Committee of Sun Yat-sen University approved all the experimental procedures (approval number # L102012020010L). The mice were randomly divided into groups (*n* = 6 /group).

In the experiments shown in Figs. 4, 5, female C57BL/6 J mice (6–8 weeks old) were injected subcutaneously with MC38-Vector (Vec.)/Scramble (Scr.), MC38-USP14/Scr., and MC38-USP14/shIDO1 cells (5 × 10^5^ cells/mouse) at day 0 (Fig. 4). Female C57BL/6 J mice (6–8 weeks old) were injected subcutaneously with MC38-Vec./Scr., MC38-shUSP14/Vec., MC38-shUSP14/USP14, and MC38-shUSP14/C114A cells (5 × 10^5^ cells/mouse) at day 0 (Fig. 5). At day 21 after inoculation, mouse plasma was collected and subjected to HPLC-MS/MS analysis to generate the tryptophan metabolomic profiles, and the tumors were excised for flow cytometry or immunofluorescence analyses.

In the experiments shown in Supplementary Fig. 5l, female C57BL/6 J mice (6–8 weeks old) were injected subcutaneously with MC38 cells (5 × 10^5^ cells/mouse) at day 0. Mice were intraperitoneally injected with Vehicle (2% dimethyl sulfoxide (DMSO) + 30% polyethylene glycol (PEG)300 + 2% Tween 80), KYN (200 µм kg^−1^/day; #HY-104026; MedChem Express (MCE), Monmouth Junction, NJ, USA), IU1 (50 µм kg^−1^/day; #S7134; Selleck, Houston, TX, USA) or Epacadostat (100 µм kg^−1^/day; #HY-15689; MCE) at day 0, and the experiment lasted for 10 days. Mice were sacrificed on day 21, and their tumors were excised and fixed in formalin, embedded in paraffin, and subjected to immunofluorescence analysis.

In the experiments shown in Supplementary Fig. 5m-o, female C57BL/6 J mice (6–8 weeks old) were injected subcutaneously with MC38 cells (5 × 10^5^ cells/mouse) at day 0. Mice were intraperitoneally injected with Vehicle (2% DMSO + 30% PEG300 + 2% Tween 80), IU1 (40 mg kg^−1^/day), or Epacadostat (40 mg kg^−1^/day) every day, and the experiment lasted for 21 days.

In the experiments shown in Fig. 6, female C57BL/6 J mice (6–8 weeks old) were injected subcutaneously with MC38-Scramble and MC38-shUSP14 (5 × 10^5^ cells/mouse) at day 0, and then treated with anti-PD-1 or IgG isotype control. Female C57BL/6 J mice (6–8 weeks old) were injected subcutaneously with MC38 cells (5 × 10^5^ cells/mouse) at day 0, and then treated with anti-PD-1, IU1, anti-PD-1 plus IU1, or isotype control. Anti-PD-1 monoclonal antibody (BE0146; clone RMP1-14; Bio-Xcell, Lebanon, NH, USA; diluted with phosphate-buffered saline (PBS)) or IgG isotype control (BE0089; clone 2A3; Bio-Xcell; diluted with PBS) was administered on days 3, 5, 7, 10, 12, 14, 17, 19, and 21 post-tumor inoculations by intraperitoneal injection at a dosage of 200 µg/injection. Mice were intraperitoneally injected with Vehicle (2% DMSO + 30% PEG300 + 2% Tween 80) or IU1 (40 mg kg^−1^/day) every day, and the experiment lasted for 21 days.

Tumors were measured by using an in vivo imaging system (IVIS) or calipers twice weekly. The tumor volume was calculated using the equation length × width^2^ × 0.5. Mice were generally sacrificed when tumors became necrotic, or their volume reached ~2000 mm^3^, recorded as death for the survival curve. According to the guidelines (GB/T 35892-2018), the ethics committee specified that the maximal tumor burden is no more than 10% of the body weight of animals and the average diameter is less than 20 mm. During the experiment, the tumor sizes of the mice complied with the regulations.

### Patient information and tissue specimens

We collected 119 colorectal cancer specimens that were histopathologically diagnosed at the Sun Yat-sen University Cancer Center from 2008 to 2015. All patients eligible for this study accepted surgery and were followed up regularly. The clinicopathological characteristics, including details of the covariate-related population characteristics of human research participants (such as age, gender, etc.), are summarized in Supplementary Table 1. Patients provided written informed consent, and the study was carried out according to the Declaration of Helsinki. Ethics approval (#GZR2021-254) was obtained from the Institutional Research Ethics Committee of Sun Yat-sen University Cancer Center to use the clinical specimens for research purposes.

### Cell lines and viral infection

The human colorectal carcinoma cell lines Caco2, DLD1, HCT15, HT29, LOVO, HCT116, and SW480 were purchased from ATCC (Manassas, VA, USA) and cultured in Roswell Park Memorial Institute (RPMI)1640 medium (Thermo Scientific, Waltham, MA, USA) with 10% fetal bovine serum (FBS) (Gibco, Grand Island, NY, USA), except that human tumor cell line HCT116 was grown in McCoy’s 5 A medium with 10% fetal bovine serum (FBS). The MC38 cell line (C57BL/6 J mouse colon adenocarcinoma cells) was kindly gifted by Dr. Xiaojun Xia at the State Key Laboratory of Oncology in South China, Sun Yat-Sen University Cancer Center, Guangzhou, China, originally purchased from Kerafast (Boston, MA, USA). Human normal colonic epithelial cells (NCM460) and human embryonic kidney cells (HEK293FT) were obtained from the Cell Bank of Shanghai Institutes of Biological Sciences (Shanghai, China) and cultured in Dulbecco’s modified Eagle’s medium (DMEM) with 10% FBS. All cell lines used in this study were tested to confirm that they were free of mycoplasma contamination. Human and mouse *USP14* and *IDO1* cDNAs were PCR-amplified and cloned into the pLVX-IRES-puro vector. The shRNAs in vector pSuper-neo were purchased from Transheep Bio (Shanghai, China). Cells (2 × 10^5^) were seeded and infected by retrovirus generated by pLVX-IRES-puro-cDNAs or produced by pSuper-neo-shRNAs for 3 days. All cells were infected with a pMSCV-neo-luciferase retrovirus and selected using 0.5 µg mL^−1^ puromycin and 250 µg mL^−1^ G418 for 7 days to establish stable cell lines. The indicated sequences are provided in Supplementary Table 4.

### Immunohistochemistry (IHC) and the Immunocore

Tumor tissues from mouse models were collected and fixed in 10% formalin overnight and embedded in paraffin. Immunohistochemistry analysis was performed on 119 paraffin-embedded CRC tissues following the manufacturer’s protocols. Anti-IDO1 Rabbit antibody, anti-USP14 Rabbit antibody, anti-Kynurenine Mouse antibody, and anti-cleaved Caspase-3 Rabbit antibody were used for IHC staining. IHC slides were scanned using a Vectra Polaris Scanner (Akoya Biosciences, Marlborough, MA, USA) at ×20 magnification. IHC staining of IDO1, USP14, and KYN were scored separately by two independent pathologists. The IHC staining for IDO1, USP14, and KYN was graded with four scores: strong, +3; moderate, +2; weak, +1; and absent, 0. An optimal threshold was then determined to define tumors with high expression using a receiver operating characteristic (ROC) curve. Specimens with scores of +3 or +2 were defined as IDO1 or USP14 high, and those with scores of +1 or 0 were defined as IDO1 or USP14 low. Specimens with scores of +3 were defined as KYN high, and those with scores of +2, +1, or 0 were defined as low.

After immunohistochemical staining of each specimen with anti-CD3 and anti-CD8, all slides were scanned at an absolute magnification of × 20 (resolution of 0.5 μm per pixel). Digital image analysis (DIA) of the whole slide images was performed using HALO version 3.2.1851 (Indica Labs, Corrales, NM, USA). Cancer-specific artificial intelligence tissue classifiers were trained using the HALO AI module to segment the tumor center, invasion margin, stroma, and background (consisting of necrosis, artifacts, and glass)^68^. Subsequently, the CytoNuclear Algorithm v2.0.9 in HALO was used to detect cytoplasmic CD8^+^ and CD3^+^ cells in the tumor center and invasion margin based on cytonuclear features. The density (number of positive cells per mm^2^) of CD3- and CD8-positive cells in the colon tumor center and invasion margin were calculated^32^, and the four values obtained were converted into percentiles. The mean of the four percentiles was calculated and converted into an Immunoscore in a three-category Immunoscore analysis: low (0–25%), intermediate (25–70%), and high (70–100%)^32^.

### Immunofluorescence

Paraffin-embedded samples were sectioned at 4 mm thickness. Antigen retrieval was performed in a microwave oven (95 °C, 30 min) in citrate solution. For the cell samples, cells (5 × 10^4^) were seeded on coverslips and fixed for 15–20 min using fresh, methanol-free 4% formaldehyde. The slides or the coverslips were then blocked in PBS containing 2% bovine serum albumin for 1 h at room temperature. For dual immunofluorescence staining, the slides or the coverslips were incubated in a mixture of two primary antibodies overnight at 4 °C. The following primary antibodies were used: anti-CD8 Rat antibody, anti-Granzyme B Mouse antibody, anti-CD4 Rat antibody, anti-FOXP3 Rabbit antibody, anti-IDO1 Rabbit antibody, anti-USP14 Mouse antibody, and anti-Kynurenine Mouse antibody. The slides or coverslips were washed using cold PBS and incubated with a mixture of two secondary antibodies raised in different species for 1 h at room temperature in the dark. The following secondary antibodies were used: Alexa Fluor 488-labeled anti-Rabbit, Alexa Fluor 594-labeled anti-Rat, Alexa Fluor 488-labeled anti-Mouse, and Alexa Fluor 594-labeled anti-Mouse. The slides and coverslips were counterstained using 4′,6-diamidino-2-phenylindole (DAPI) (Sigma–Aldrich, St. Louis, MO, USA) to visualize the nuclei. Each sample was examined under a fluorescence microscope.

### Multiple immunofluorescence analysis

For multiple-color staining, an Opal^TM^ 5-color IHC kit was used according to the manual provided (Akoya Biosciences). Paraffin-embedded samples were sequentially stained with primary antibodies and horseradish peroxidase (HRP)-conjugated secondary antibodies. One of the four Opal reagents was used for staining, followed by microwave treatment and another round of staining. Anti-CD3 Rabbit antibody, anti-CD8 Mouse antibody, anti-CD4 Rabbit antibody, anti-IDO1 Rabbit antibody, anti-USP14 Rabbit antibody, and anti-Keratin Mouse antibody were used as primary antibodies. The dyes Opal520, Opal570, Opal650, Opal480, and DAPI were used for staining. Samples were visualized using the Vectra Polaris Automated Quantitative Pathology Imaging System (Perkin-Elmer, Waltham, MA, USA).

All slides were scanned at an absolute magnification of × 20 (resolution of 0.5 μm per pixel). Digital image analysis (DIA) of the whole slide images was performed using HALO version 3.2.1851. Cancer-specific artificial intelligence tissue classifiers were trained using the HALO AI module to segment the tumor center, invasion margin, stroma, and background (consisting of necrosis, artifacts, and glass). Subsequently, the HighPlex FL Algorithm v4.0.4 in HALO was used to detect CD3^+^ and CD3^+^CD8^+^ cells based on cytonuclear features. The density (number of positive cells per mm^2^) of CD3^+^ and CD3^+^CD8^+^ cells on the slides was calculated.

### Immunoprecipitation (IP) assays

Cell lysates were prepared from the indicated cells using lysis buffer (150 mм NaCl, 10 mм HEPES, pH 7.4, 1% NP-40). The lysates were then incubated with anti-USP14 Rabbit antibody, or anti-IDO1 Rabbit antibody, or anti-TRIM21 Rabbit antibody, and protein G-conjugated agarose, or Flag, HA affinity agarose (Sigma–Aldrich), at 4 °C overnight. Beads containing affinity-bound proteins were washed six times using IP wash buffer (150 mм NaCl, 10 mм HEPES, pH 7.4, 0.1% NP-40), followed by elution using 1 м glycine (pH 3.0). The eluates were then mixed with sample buffer, denatured, and subjected to western blotting analysis. HA-IDO1-specific bands were subjected to MS analysis. GST fusion proteins were prepared following a standard protocol. For the in vitro binding assays, IDO1-GST fusion proteins bound to GSH-Sepharose (Sigma–Aldrich) were incubated with cell lysates. After washing, the bound proteins were separated by SDS-PAGE and subjected to Coomassie blue staining.

### Mass spectrometry (MS) analysis

To identify the binding proteins of IDO1, HCT116 and SW480 cells were transfected with HA-IDO1 or pLVX-IRES-vector. Lysates were immunoprecipitated with HA-beads. Beads containing affinity-bound proteins were washed six times using wash buffer (150 mм NaCl, 10 mм HEPES, pH 7.4, 0.1% NP-40), followed by elution using 1 м glycine (pH 3.0). Elutes were subjected to mass spectrometry (MS). The mass spectrometry data were deposited in the iProX database (#PXD033718). Information on the peptides and counts for IDO1-binding proteins analyzed by IP/MS assays and a full list of the enriched pathways of IDO1-interacting proteins are provided as Supplementary Data 1, 2.

### The detection assays of tryptophan-associated metabolites

Cells (2 × 10^6^/100 mm dish) were cultured in the presence or absence of KYNU (1 μм) for 24 h. Media were removed and replaced with 10 mL of serum-free DMEM. Supernatants were collected 48 h later, and any floating cells were removed by 0.45 mm filtration. The amount of KYN and TRP in the supernatant was determined using a kynurenine/tryptophan ratio ELISA pack (ISE-2227; ImmuSmol, Bordeaux, France). Tryptophan (100 µм) was added to the DMEM previously. All experiments were performed according to the manufacturer’s instructions. The TRP and KYN values were extrapolated from respective known standards provided in each kit, and the numeric ratio (KYN/TRP) was calculated and plotted using GraphPad prism 7 (GraphPad Inc., La Jolla, CA, USA). The quantitative levels of KYN and TRP are summarized in Supplementary Data 3, 5.

For mouse plasma samples, 100 ± 5 μL of mouse plasma was placed in a 2 mL centrifuge tube after being thawed on ice. We added 10 μL of internal standard solution and 0.4 mL acetonitrile-methanol, and then mixed the samples by vortexing. After centrifugation at 14,000 × *g*, 400 μL of the supernatant was collected and dried under nitrogen gas. The residue was dissolved in 100 μL of acetonitrile-water and then centrifuged at 14,000 × *g*. The supernatant was then injected into the HPLC-MS/MS apparatus for analysis by the Applied Protein Technology Company (Shanghai, China). MultiQuant or Analyst was used for quantitative data processing. The integration was further checked manually. The quantitative results are summarized in Supplementary Data 4, 6 and the standard curve of metabolites of tryptophan metabolism is provided in Supplementary Data 7.

### Luciferase assays

AhR activation was measured using an AhR-dependent luciferase reporter (pXRE4-SV40-Luc). HCT116 and SW480 cells were transfected using Lipofectamine 3000 reagent (#L3000015; Invitrogen, Waltham, MA, USA) according to the manufacturer’s recommendations, together with a Renilla luciferase construct control vector (pRLTK; Promega) to which firefly values were normalized. Cells were treated with DMSO, 10 nм TCDD (#48599; Sigma–Aldrich), and the indicated inhibitors for 24 h. Luciferase and Renilla signals were measured 24 h after transfection using the Dual Luciferase Reporter Assay Kit (#E1960; Promega, Madison, WI, USA).

### Quantitative real-time reverse transcription PCR (qRT-PCR)

Total RNA was isolated using the TRIzol reagent (#15596026; Invitrogen) and reverse-transcribed using M-MLV Reverse Transcriptase (#M1701; Promega). The resulting cDNA was subjected to qRT-PCR analysis, performed in triplicate, using the TB Green Fast qPCR Mix (#RR430A; Takara, Dalian, China) on a CFX96 Real-Time System C1000 Cycler (Bio-Rad Laboratories, Singapore). PCR product specificity was confirmed by melting-curve analysis. The relative mRNA expression was calculated as 2^[(Ct of gene) − (Ct of GAPDH)]^, in which Ct represents the cycle threshold for each transcript. The primer sequences used in the PCR reactions are listed in Supplementary Table 4.

### Isolation of the nuclear extract

The nuclear extract was prepared using an NE-PER Nuclear Cytoplasmic Extraction Reagent kit (Pierce, Rockford, IL, USA) according to the manufacturer’s instructions. Briefly, the treated cells were washed twice with PBS and centrifuged at 500 × *g* for 5 min. The cell pellet was suspended in 200 μL of cytoplasmic extraction reagent I by vortexing. The suspension was incubated on ice for 10 min followed by the addition of 11 μL of cytoplasmic extraction reagent II, vortexed for 5 s, incubated on ice for 1 min, and centrifuged for 5 min at 16,000 × *g*. The resulting supernatant, constituting the cytoplasmic extract, was used for subsequent experiments. The insoluble pellet fraction, which contained crude nuclei, was resuspended in 100 μL of nuclear extraction reagent by vortexing for 15 s, incubated on ice for 10 min, and then centrifuged for 10 min at 16,000 × *g*. The resulting supernatant, constituting the nuclear extract, was used for subsequent experiments.

### Western blotting analysis

The protein concentration was determined using a bicinchoninic acid (BCA) assay according to the manufacturer’s instructions. Western blotting analyses were performed according to a standard protocol using the following antibodies: anti-IDO1 Mouse antibody, anti-USP14 Rabbit antibody, anti-TRIM21 Rabbit antibody, anti-GAPDH Rabbit antibody, anti-HA Rabbit antibody, anti-MYC Mouse antibody, anti-Flag Rabbit antibody, anti-ubiquitin Rabbit antibody, anti-Aryl hydrocarbon Receptor Mouse monoclonal antibody, anti-tubulin Rabbit antibody, anti-TopBP1 Rabbit antibody, and the secondary antibodies goat anti-Rabbit immunoglobulin G and goat anti-mouse immunoglobulin G. Western blotting grayscale analyses were performed using Image J 1.42q software (NIH, Bethesda, MD, USA). The levels of IDO1 protein were quantified by determining the gray level of each band using Image J and normalized using GAPDH. Information on the antibodies is provided in Supplementary Table 5. Unprocessed scans of immunoblots are provided as Source Data.

### In vitro ubiquitination assay

Flag-IDO1 was expressed in HEK293FT cells. After Flag-agarose pulldown, Flag-IDO1 was eluted using elution buffer containing the Flag peptide. The eluates were incubated together at 30 °C in a 25 μL reaction mixture containing 20 mм HEPES buffer (pH 7.4), 10 mм MgCl_2_, 1 mм DTT, 1 mM ATP, 5 mм creatine phosphate, 1 U creatine kinase, 2 μм ubiquitin, 50 nм ubiquitin-like modifier activating enzyme 1 (UBE1), and 500 nм ubiquitin-conjugating enzyme E2 N (UBE2N) or ubiquitin-conjugating enzyme E2 D2 (UBE2D2) for 3 h. The reaction was stopped by adding 2 × loading buffer, boiled for 10 min, and subjected to immunoblotting.

### Single-cell isolation and flow cytometry assay

Surface and intracellular staining cocktail master mixes were prepared prior to each experiment. In brief, CRC tumor single cells were isolated using the mouse Tumor Dissociation Kit (#130-096-730; Miltenyi Biotec, Bergisch Gladbach, Germany) following a standard protocol. Digested tumors were then mashed through 40 mm filters into RPMI-1640 and centrifuged at 300 × *g* for 5 min at 4 °C. All single cells were depleted of erythrocytes using Red Cell lysis buffer for 1 min at room temperature. 2 × 10^6^ or fewer cells per tumor were blocked with Blocking Reagent for 10 min and incubated with the surface antibody mix for 30 min at room temperature. Cells were washed once with PBS and incubated with Dead Cell Dyes at 2.5 mм for 3 min for viability staining. Cells were washed with Cell Staining Buffer. For intracellular staining, cells were incubated with FOXP3 Fixation/Permeabilization working solution by diluting Fixation/Permeabilization Concentrate with Fixation/Permeabilization Diluent at 4 °C overnight (in the dark). Cells were washed twice with a working solution of Permeabilization Buffer. Cells were incubated with the intracellular antibody mix for 1 h at room temperature and then washed twice with Cell Staining Buffer. Cells were resuspended in 500 µL PBS. Samples were analyzed using a flow cytometer (cytoFLEX LX, Beckman Coulter, Indianapolis, IN, USA) with CytExpert software (Beckman Coulter). Percentages of each cell population were analyzed by FlowJo software (Tree Star, Ashland, OR, USA) and GraphPad Prism 7 software. Flow cytometry staining panels are detailed in Supplementary Table 6.

### Isolation of T cells and flow cytometry assay

CD8^+^ and CD4^+^ T cells were isolated from single-cell suspensions using a mouse CD8a (Ly-2) MicroBeads kit (#130-117-044; Miltenyi Biotec) and a mouse CD4 (L3T4) MicroBeads kit (#130-117-043; Miltenyi Biotec) respectively. Isolation was performed according to the manufacturer’s recommendation. A total of 2 × 10^6^ cells were blocked with Blocking Reagent for 10 min and incubated with anti-Granzyme B or anti-CD25/anti-FOXP3 for 30 min at room temperature. Cells were washed twice with Cell Staining Buffer and resuspended in 500 µL PBS. Samples were analyzed with a flow cytometry. Percentages of each cell population were analyzed by FlowJo and GraphPad Prism 7 software.

### T-cell suppression assay

Unstimulated T cells were used as a negative control. Naive CD8^+^ lymphocytes were magnetically isolated from single-cell suspensions that were prepared from the spleens of tumor-free C57BL/6 J mice using a mouse Spleen Dissociation Kit (#130-095-926; Miltenyi Biotec) and mouse CD8a (Ly-2) MicroBeads (#130-117-044; Miltenyi Biotec). CD8^+^ T cells were used immediately after isolation. Naive CD8^+^ T cells were stained with 5 mM of carboxyfluorescein succinimidyl ester (CFSE) by incubation at room temperature for 5 min in the dark. The CFSE surplus was removed by two washing steps with 1 × PBS. Enriched naive CD8^+^ T cells (1 × 10^5^) were stimulated in U-bottomed 96-well plates that were precoated with 2 μg mL^−1^ anti-CD3 (#130-097-621; Miltenyi Biotec) and anti-CD28 (#130-093-182; Miltenyi Biotec)^69^. After being stimulated for 48 h, the cells were transferred to new wells and rested for 24 h.

For the conditioned medium, tumor cells were seeded at a density of 50,000 cells/cm^2^ and cultured in the presence or absence of KYNU (1 μм) or vehicle control (10% DMSO in PBS) for 48 h. Supernatants of each group of tumor cells were centrifuged at 1000 × *g* for 5 min. The CFSE-labeled CD8^+^ T cells were exposed to a conditioned medium or DMEM supplemented with 5% FBS and cultured for 5 days. Then, the CD8^+^ T cells were collected, washed twice in 1 × PBS, and resuspended in 500 µL of 1 × PBS before analysis by flow cytometry. Unstimulated T cells were used as a negative control. Measurements were performed on a flow cytometer (Beckman Coulter CytoFLEX LX) with CytExpert software, and CFSE intensity was quantified from the peaks identified by flow cytometry. CFSE peaks indicated the division times. Division times ≤1, and ≥2 were defined as low proliferation and high proliferation, respectively. The supernatant medium was used to quantify IFN-γ production by ELISA following the manufacturer’s manual (#430804; Biolegend, San Diego, CA, USA).

### Statistics

Statistical analysis was performed using GraphPad Prism 7 or SPSS 21.0 (IBM Corp., Armonk, NY, USA). For comparison of two groups, *P-*values were calculated using a two-sided Student’s *t*-test. For comparison of more than two groups, *P*-values were calculated using ANOVA. The relationship between USP14 or IDO1 expression and clinicopathological characteristics was tested using a two-sided χ^2^ test. Survival curves were plotted using the Kaplan–Meier method and compared by the log-rank test. Multivariate survival analyses using Cox proportional hazard regression models were performed to evaluate independent prognostic factors. *P* < 0.05 was considered statistically significant in all cases.

### Reporting summary

Further information on research design is available in the Nature Research Reporting Summary linked to this article.

## Supplementary information

Supplementary Information

reporting summary

Description of Additional Supplementary Files

Supplementary Data 1

Supplementary Data 2

Supplementary Data 3

Supplementary Data 4

Supplementary Data 5

Supplementary Data 6

Supplementary Data 7

## Data Availability

All the other data supporting the findings of this study are available within the article and its Supplementary information files. The mass spectrometry proteomics data have been deposited to the ProteomeXchange Consortium (http://proteomecentral.proteomexchange.org) via the iProX partner repository with the dataset identifier PXD033718. A reporting summary for this article is available as a Supplementary Information file. The main data supporting the findings of this study are available within the article and its Supplementary Figures. Specific data *P-*values are also included within the Source Data file. Source data are provided with this paper.

## Source data

Source Data

## References

[1] Larkin J (2015). Combined nivolumab and ipilimumab or monotherapy in untreated melanoma. N. Engl. J. Med..

[2] Borghaei H (2015). Nivolumab versus docetaxel in advanced nonsquamous non-small-cell lung cancer. N. Engl. J. Med..

[3] Zhou C (2021). Outcomes and toxicities of immune checkpoint inhibitors in colorectal cancer: a real-world retrospective analysis. Cancer Commun. (Lond.).

[4] Sharma P, Hu-Lieskovan S, Wargo JA, Ribas A (2017). Primary, adaptive, and acquired resistance to cancer immunotherapy. Cell.

[5] Frumento G (2002). Tryptophan-derived catabolites are responsible for inhibition of T and natural killer cell proliferation induced by indoleamine 2,3-dioxygenase. J. Exp. Med.

[6] Munn DH (1999). Inhibition of T cell proliferation by macrophage tryptophan catabolism. J. Exp. Med..

[7] Mellor AL, Keskin DB, Johnson T, Chandler P, Munn DH (2002). Cells expressing indoleamine 2,3-dioxygenase inhibit T cell responses. J. Immunol..

[8] Holmgaard RB (2015). Tumor-expressed IDO recruits and activates MDSCs in a Treg-dependent manner. Cell Rep..

[9] Rothhammer V, Quintana FJ (2019). The aryl hydrocarbon receptor: an environmental sensor integrating immune responses in health and disease. Nat. Rev. Immunol..

[10] Cheong JE, Sun L (2018). Targeting the IDO1/TDO2-KYN-AhR pathway for cancer immunotherapy - challenges and opportunities. Trends Pharmacol. Sci..

[11] Prendergast GC (2011). Cancer: why tumours eat tryptophan. Nature.

[12] Liu Y (2018). Tumor-repopulating cells induce PD-1 expression in CD8(+) T cells by transferring kynurenine and AhR activation. Cancer Cell.

[13] de Araújo EF (2017). The IDO-AhR axis controls Th17/Treg immunity in a pulmonary model of fungal infection. Front. Immunol..

[14] Brandacher G (2006). Prognostic value of indoleamine 2,3-dioxygenase expression in colorectal cancer: effect on tumor-infiltrating T cells. Clin. Cancer Res..

[15] Holmgaard RB, Zamarin D, Munn DH, Wolchok JD, Allison JP (2013). Indoleamine 2,3-dioxygenase is a critical resistance mechanism in antitumor T cell immunotherapy targeting CTLA-4. J. Exp. Med..

[16] Komiya T, Huang CH (2018). Updates in the clinical development of epacadostat and other indoleamine 2,3-dioxygenase 1 inhibitors (IDO1) for human cancers. Front. Oncol..

[17] Mitchell TC (2018). Epacadostat plus pembrolizumab in patients with advanced solid tumors: phase I results from a multicenter, open-label phase I/II trial (ECHO-202/KEYNOTE-037). J. Clin. Oncol..

[18] Gunther J, Dabritz J, Wirthgen E (2019). Limitations and off-target effects of tryptophan-related IDO inhibitors in cancer treatment. Front. Immunol..

[19] Long GV (2019). Epacadostat plus pembrolizumab versus placebo plus pembrolizumab in patients with unresectable or metastatic melanoma (ECHO-301/KEYNOTE-252): a phase 3, randomised, double-blind study. Lancet Oncol..

[20] Moyer BJ (2017). Indoleamine 2,3-dioxygenase 1 (IDO1) inhibitors activate the aryl hydrocarbon receptor. Toxicol. Appl. Pharmacol..

[21] Metz R (2012). IDO inhibits a tryptophan sufficiency signal that stimulates mTOR: A novel IDO effector pathway targeted by D-1-methyl-tryptophan. Oncoimmunology.

[22] Murray IA, Patterson AD, Perdew GH (2014). Aryl hydrocarbon receptor ligands in cancer: friend and foe. Nat. Rev. Cancer.

[23] Timosenko E (2016). Nutritional stress induced by tryptophan-degrading enzymes results in ATF4-dependent reprogramming of the amino acid transporter profile in tumor cells. Cancer Res..

[24] Zhai L (2015). Molecular pathways: targeting IDO1 and other tryptophan dioxygenases for cancer immunotherapy. Clin. Cancer Res..

[25] Hennequart M (2017). Constitutive IDO1 expression in human tumors is driven by cyclooxygenase-2 and mediates intrinsic immune resistance. Cancer Immunol. Res..

[26] Kim NS, Torrez T, Langridge W (2019). LPS enhances CTB-INSULIN induction of IDO1 and IL-10 synthesis in human dendritic cells. Cell. Immunol..

[27] Babcock TA, Carlin JM (2000). Transcriptional activation of indoleamine dioxygenase by interleukin 1 and tumor necrosis factor alpha in interferon-treated epithelial cells. Cytokine.

[28] Broekhuizen M (2020). l-Tryptophan-induced vasodilation is enhanced in preeclampsia: studies on its uptake and metabolism in the human placenta. Hypertension (Dallas, Tex.: 1979).

[29] Orabona C (2008). SOCS3 drives proteasomal degradation of indoleamine 2,3-dioxygenase (IDO) and antagonizes IDO-dependent tolerogenesis. Proc. Natl Acad. Sci. USA.

[30] Thomas SR (2007). Post-translational regulation of human indoleamine 2,3-dioxygenase activity by nitric oxide. J. Biol. Chem..

[31] Lou Q (2019). miR-448 targets IDO1 and regulates CD8(+) T cell response in human colon cancer. J. Immunother. Cancer.

[32] Pagès F (2018). International validation of the consensus Immunoscore for the classification of colon cancer: a prognostic and accuracy study. Lancet (Lond., Engl.).

[33] Lee BH (2016). USP14 deubiquitinates proteasome-bound substrates that are ubiquitinated at multiple sites. Nature.

[34] Lee BH (2010). Enhancement of proteasome activity by a small-molecule inhibitor of USP14. Nature.

[35] Pan JA (2016). TRIM21 ubiquitylates SQSTM1/p62 and suppresses protein sequestration to regulate redox homeostasis. Mol. Cell.

[36] Gao J (2016). Loss of IFN-γ pathway genes in tumor cells as a mechanism of resistance to anti-CTLA-4 therapy. Cell.

[37] Liu M (2018). Targeting the IDO1 pathway in cancer: from bench to bedside. J. Hematol. Oncol..

[38] Muller AJ (2008). Chronic inflammation that facilitates tumor progression creates local immune suppression by inducing indoleamine 2,3 dioxygenase. Proc. Natl Acad. Sci. USA.

[39] Prendergast GC, Mondal A, Dey S, Laury-Kleintop LD, Muller AJ (2018). Inflammatory reprogramming with IDO1 inhibitors: turning immunologically unresponsive ‘Cold’ tumors ‘Hot’. Trends Cancer.

[40] Smith C (2012). IDO is a nodal pathogenic driver of lung cancer and metastasis development. Cancer Discov..

[41] Mondanelli, G. et al. A novel mutation of indoleamine 2,3-dioxygenase 1 causes a rapid proteasomal degradation and compromises protein function. *J. Autoimmunity*10.1016/j.jaut.2020.102509 (2020). 10.1016/j.jaut.2020.102509 32605792

[42] Fujigaki H, Seishima M, Saito K (2012). Posttranslational modification of indoleamine 2,3-dioxygenase. Anal. Bioanal. Chem..

[43] Albini E (2017). Distinct roles of immunoreceptor tyrosine-based motifs in immunosuppressive indoleamine 2,3-dioxygenase 1. J. Cell. Mol. Med..

[44] Chen M (2016). TRIM14 inhibits cGAS degradation mediated by selective autophagy receptor p62 to promote innate immune responses. Mol. Cell.

[45] Zhang Z (2013). The E3 ubiquitin ligase TRIM21 negatively regulates the innate immune response to intracellular double-stranded DNA. Nat. Immunol..

[46] Jung H (2013). Deubiquitination of Dishevelled by Usp14 is required for Wnt signaling. Oncogenesis.

[47] Meng Q (2015). Reversible ubiquitination shapes NLRC5 function and modulates NF-kappaB activation switch. J. Cell Biol..

[48] Liu B (2018). Proteome-wide analysis of USP14 substrates revealed its role in hepatosteatosis via stabilization of FASN. Nat. Commun..

[49] Li H, Quan J, Zhao X, Ling J, Chen W (2021). USP14 negatively regulates RIG-I-mediated IL-6 and TNF-α production by inhibiting NF-κB activation. Mol. Immunol..

[50] Li H (2019). USP14 promotes K63-linked RIG-I deubiquitination and suppresses antiviral immune responses. Eur. J. Immunol..

[51] Huang G, Li L, Zhou W (2015). USP14 activation promotes tumor progression in hepatocellular carcinoma. Oncol. Rep..

[52] Wu N (2013). Over-expression of deubiquitinating enzyme USP14 in lung adenocarcinoma promotes proliferation through the accumulation of beta-catenin. Int. J. Mol. Sci..

[53] Mialki RK, Zhao J, Wei J, Mallampalli DF, Zhao Y (2013). Overexpression of USP14 protease reduces I-κB protein levels and increases cytokine release in lung epithelial cells. J. Biol. Chem..

[54] Chen M (2020). TRIM14 promotes noncanonical NF-κB activation by modulating p100/p52 stability via selective autophagy. Adv. Sci. (Weinh., Baden.-Wurtt., Ger.).

[55] Mines MA, Goodwin JS, Limbird LE, Cui FF, Fan GH (2009). Deubiquitination of CXCR4 by USP14 is critical for both CXCL12-induced CXCR4 degradation and chemotaxis but not ERK ativation. J. Biol. Chem..

[56] Chen IX (2019). Blocking CXCR4 alleviates desmoplasia, increases T-lymphocyte infiltration, and improves immunotherapy in metastatic breast cancer. Proc. Natl Acad. Sci. USA.

[57] Palmer AL (2018). Inhibition of the deubiquitinase Usp14 diminishes direct MHC class I antigen presentation. J. Immunol. (Baltim., Md.: 1950).

[58] Xu L (2020). IU1 suppresses proliferation of cervical cancer cells through MDM2 degradation. Int. J. Biol. Sci..

[59] Ma YS (2020). Inhibition of USP14 deubiquitinating activity as a potential therapy for tumors with p53 deficiency. Mol. Ther. Oncolytics.

[60] Xia X (2019). Inhibition of USP14 enhances the sensitivity of breast cancer to enzalutamide. J. Exp. Clin. Cancer Res.

[61] Bessede A (2014). Aryl hydrocarbon receptor control of a disease tolerance defence pathway. Nature.

[62] Bunaciu RP, Yen A (2013). 6-Formylindolo (3,2-b)carbazole (FICZ) enhances retinoic acid (RA)-induced differentiation of HL-60 myeloblastic leukemia cells. Mol. Cancer.

[63] DiNatale BC (2010). Kynurenic acid is a potent endogenous aryl hydrocarbon receptor ligand that synergistically induces interleukin-6 in the presence of inflammatory signaling. Toxicol. Sci..

[64] Sadik A (2020). IL4I1 is a metabolic immune checkpoint that activates the AHR and promotes tumor progression. Cell.

[65] Hankinson O (1995). The aryl hydrocarbon receptor complex. Annu. Rev. Pharmacol. Toxicol..

[66] Soliman HH (2014). A first in man phase I trial of the oral immunomodulator, indoximod, combined with docetaxel in patients with metastatic solid tumors. Oncotarget.

[67] Lv C (2021). USP14 maintains HIF1-α stabilization via its deubiquitination activity in hepatocellular carcinoma. Cell Death Dis..

[68] Rasmusson A (2020). Immunogradient indicators for antitumor response assessment by automated tumor-stroma interface zone detection. Am. J. Pathol..

[69] Triplett TA (2018). Reversal of indoleamine 2,3-dioxygenase-mediated cancer immune suppression by systemic kynurenine depletion with a therapeutic enzyme. Nat. Biotechnol..

