# Supplementary material for: USP14 promotes tryptophan metabolism and immune suppression by stabilizing IDO1 in colorectal cancer
Source: Nat Commun. 2022 Sep 26;13:5644. doi: 10.1038/s41467-022-33285-x (PMC9513055; doi:10.1038/s41467-022-33285-x)
Supplement: Supplementary file 1 — Supplementary Information [file 41467_2022_33285_MOESM1_ESM.pdf]

Supplementary Information

USP14 Promotes Tryptophan Metabolism and Immune Suppression by Stabilizing IDO1 in Colorectal Cancer

Dongni Shi, Xianqiu Wu, Yunting Jian, Junye Wang, Guozhen Liu, Libing Song, Wenting Liao, et al.

Supplementary Figures

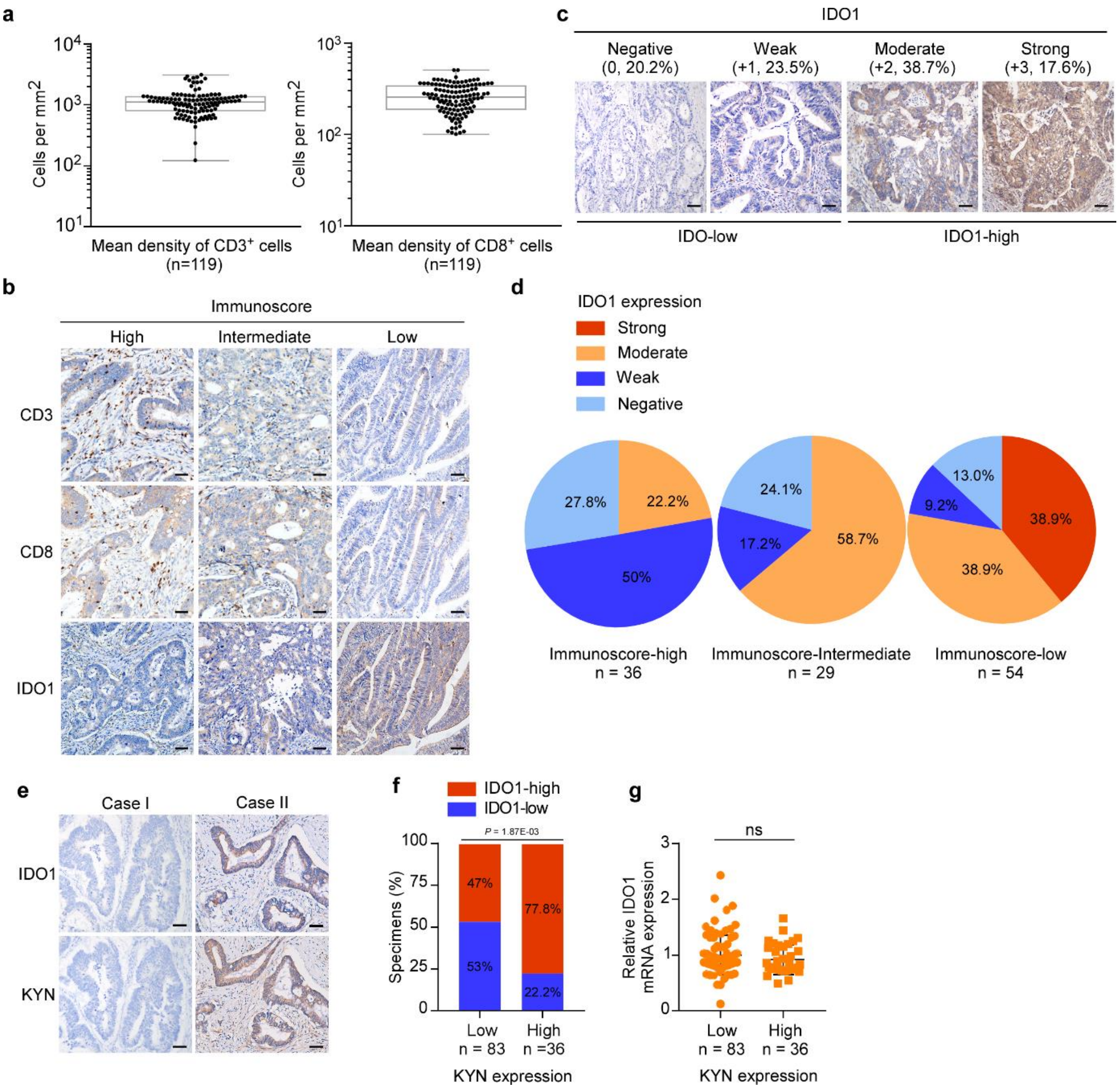

Supplementary Fig. 1. a Histogram of the mean density of CD3$^+$ and CD8$^+$ T cells in the colon tumor for 119 human CRC specimens. The lines represent the minimum, first quartile, median, third quartile and maximum of the mean density of CD3$^+$ and CD8$^+$ T cells. **b** Representative micrographs of tissue specimens with high, intermediate, or low Immunoscores stained with anti-CD3, CD8, and IDO1. Scale bars, 50 µm. **c**, **d** Percentage of samples showing negative 0, weak +1, moderate +2 and strong +3 IDO1 expression in 119 human CRC specimens relative to the Immunoscore. Weak and moderate were grouped to IDO-low. Moderate and strong were grouped to IDO1-high. Scale bars, 50 µm. **e**

Representative images of IDO1 and KYN IHC staining in human CRC specimens. Scale bars, 50 μm. **f** Correlation between IDO1 expression and KYN expression in 119 human CRC specimens. χ^2 test (two-sided). **g** The relative *IDO1* mRNA levels of 119 human CRC specimens relative to the low or high expression of KYN. ns not significant, two-sided Student's *t*-test. Source data are provided as a Source Data file.

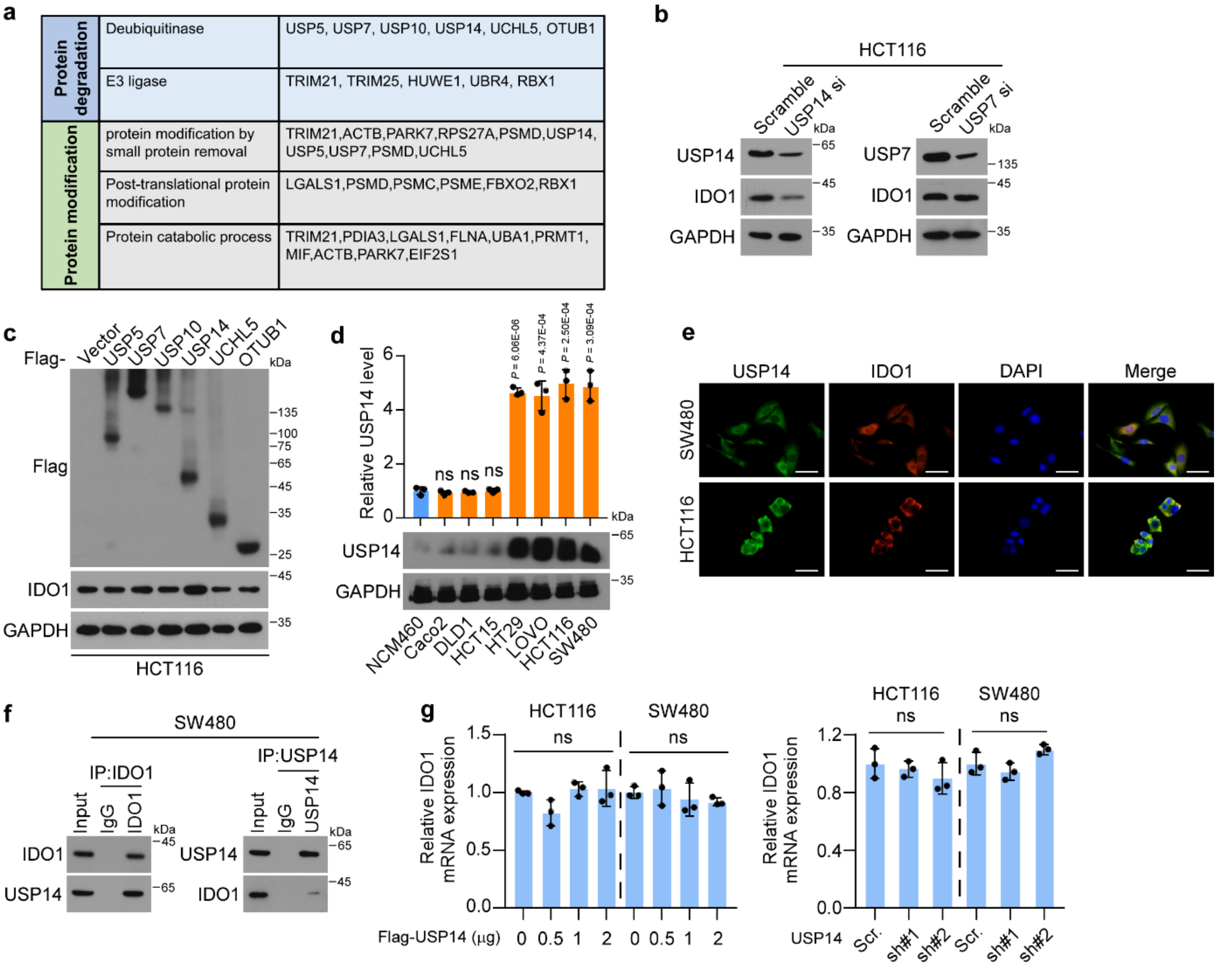

Supplementary Fig. 2. a The proteins in the list were associated with protein degradation and modification from the MS analysis results of HCT116 and SW480 cells. **b** HCT116 cells were transfected with USP14 or USP7 siRNA and analyzed with anti-IDO1 and anti-USP14 or anti-USP7. **c** HCT116 cells were transfected with plasmids encoding Flag-tagged DUBs for 48 h. Cell lysates were analyzed with anti-Flag and anti-IDO1. **d** Western blotting analysis and quantification of USP14 protein expression in the indicated cells. **e** Representative immunofluorescence images of HCT116 and SW480 cells stained with anti-USP14 and anti-IDO1. Scale bars, 50 μm. **f** SW480 cells were immunoprecipitated with anti-IDO1 or anti-USP14 and analyzed with anti-USP14 and anti-IDO1. **g** The relative *IDO1* mRNA expression in HCT116 and SW480 cells transfected with USP14 plasmids or control shRNA and *USP14* shRNA. In **d**, **g**, error bars represent the mean ± SD of three

independent experiments. ns not significant, two-sided Student's *t*-test. Source data are provided as a Source Data file.

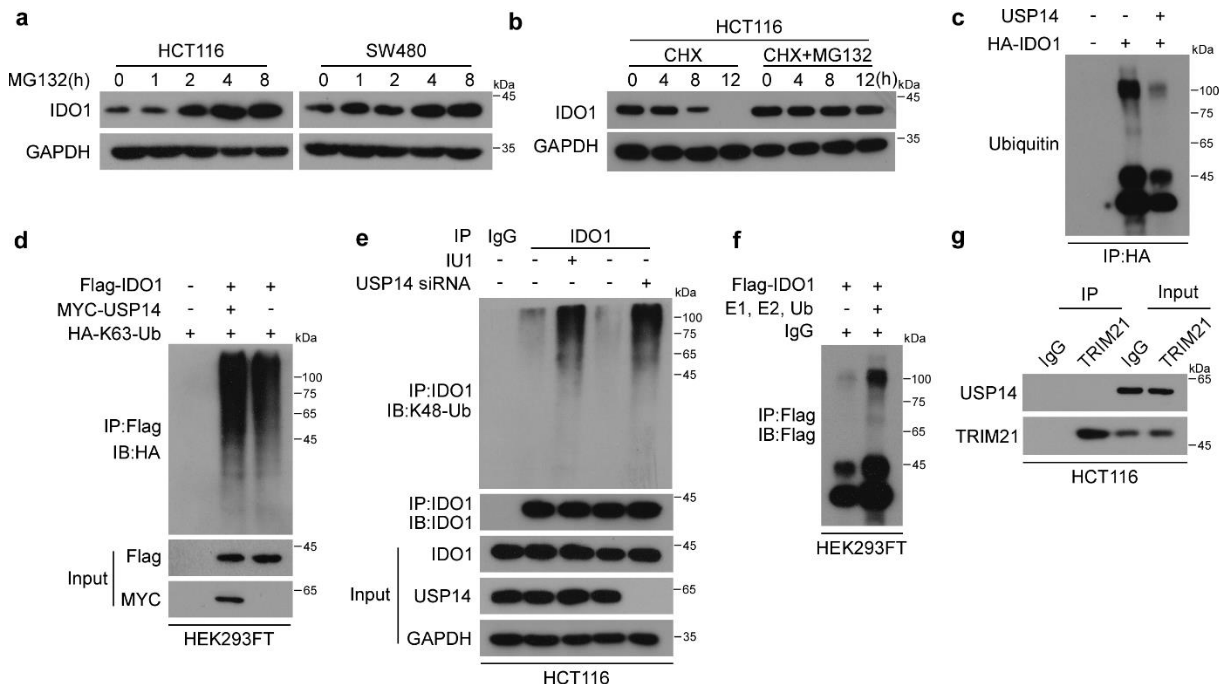

Supplementary Fig. 3. a HCT116 and SW480 cells were treated with MG132 (10 µM) for the indicated time. Cell lysates were analyzed with anti-IDO1. **b** HCT116 cells treated with CHX (0.1 mg mL^{-1}) or with CHX and MG132 (10 µM) for the indicated time and analyzed with anti-IDO1. **c** In vitro IDO1 deubiquitination assay. Purified IDO1, USP14, E1, E2, and ubiquitin were incubated for 2 h prior to western blotting analysis. **d** HEK293FT cells were transfected with HA-K63-Ub and Flag-IDO1 with or without MYC-USP14 for 48 h. Cell lysates were immunoprecipitated with anti-Flag and analyzed with the indicated antibodies. **e** HCT116 cells were transfected with USP14 siRNA for 72 h or treated with IU1 (50 µM) for 24 h, then were immunoprecipitated with anti-IDO1 and analyzed with the indicated antibodies. **f** HEK293FT cells were transfected with Flag-IDO1, and immunoprecipitated with anti-Flag and eluted using Flag peptides. The eluates were incubated with in vitro ubiquitination assay system containing E1, E2, and ubiquitin, then subjected to immunoblot. **g** HCT116 cells were immunoprecipitated with anti-TRIM21 and analyzed with anti-TRIM21 and anti-USP14. The experiment was repeated three times independently with similar results (**a-g**). Source data are provided as a Source Data file.

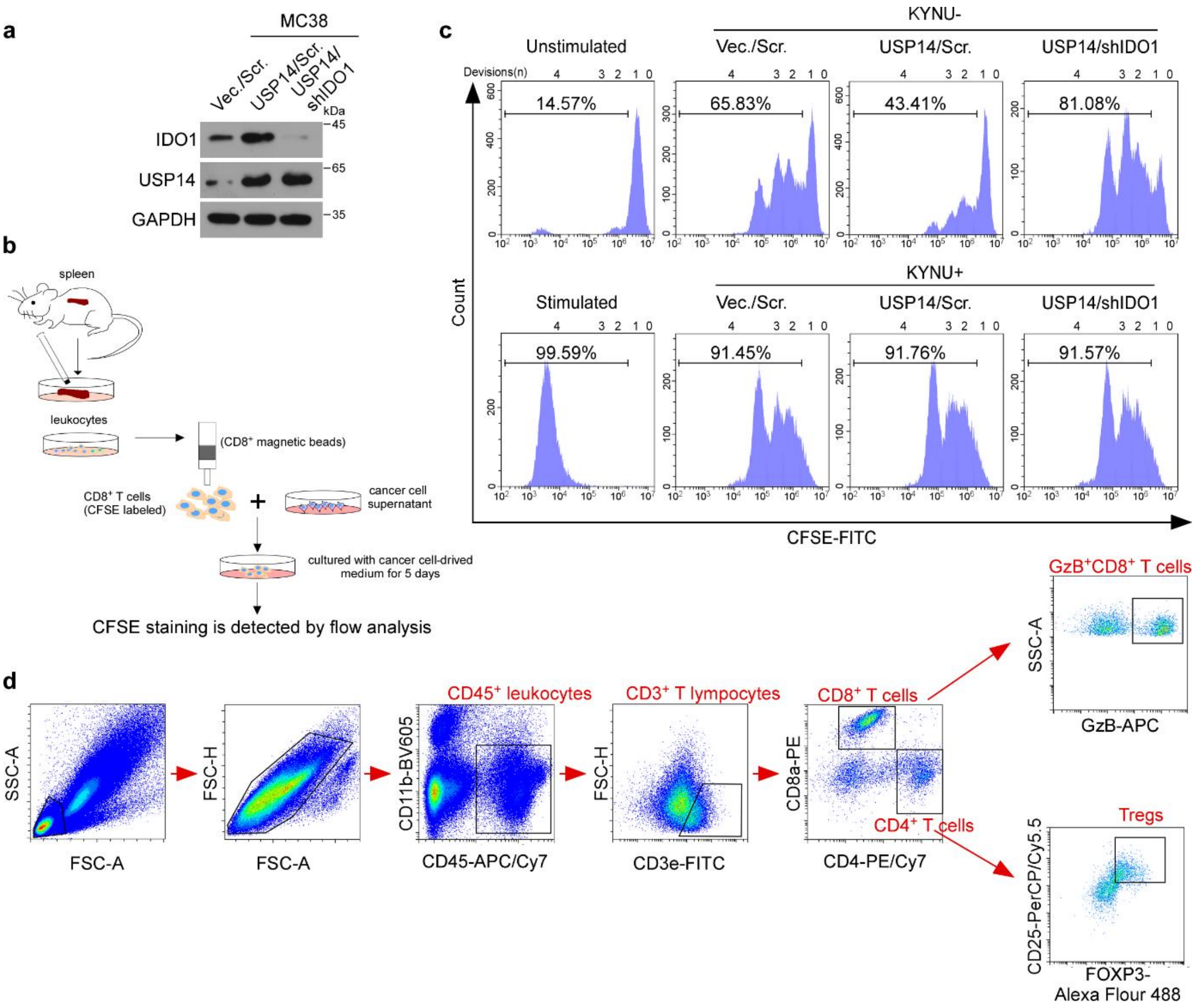

Supplementary Fig. 4. a Western blotting analysis of USP14 and IDO1 in MC38 cells reconstituted with *usp14* or *usp14* and *ido1* shRNAs. **b** Schematic of CD8$^+$ T cells isolation and standard T cell proliferation assay. **c** Representative CFSE flow cytometry histograms of the proliferation of anti-CD3/CD28 stimulated splenic CD8$^+$ T cell. Unstimulated T cells were used as negative control. Position of CFSE peaks can be used to denote the T cell division times. High and low proliferation were defined as T cell division ≥ 2 and ≤ 1, respectively (n = 3 biological replicates). **d** A flow cytometry gating strategy was designed to positively select for GzB$^+$CD8$^+$ cells and Tregs from the indicated tumors. Events were gated (black polygons) to eliminate debris and doublets, and cells were then gated for CD11b$^-$CD45$^+$ leukocytes, CD45$^+$CD3$^+$ T lymphocytes, CD45$^+$CD3$^+$CD8$^-$CD4$^+$ T helper lymphocytes, CD45$^+$CD3$^+$ CD4$^-$CD8$^+$ T lymphocytes, CD45$^+$CD3$^+$CD8$^-$CD4$^+$CD25$^+$FOXP3$^+$

regulatory T lymphocytes. $CD45^+CD3^+CD4^-CD8^+GzB^+$ T lymphocytes. GzB Granzyme B. Source data are provided as a Source Data file.

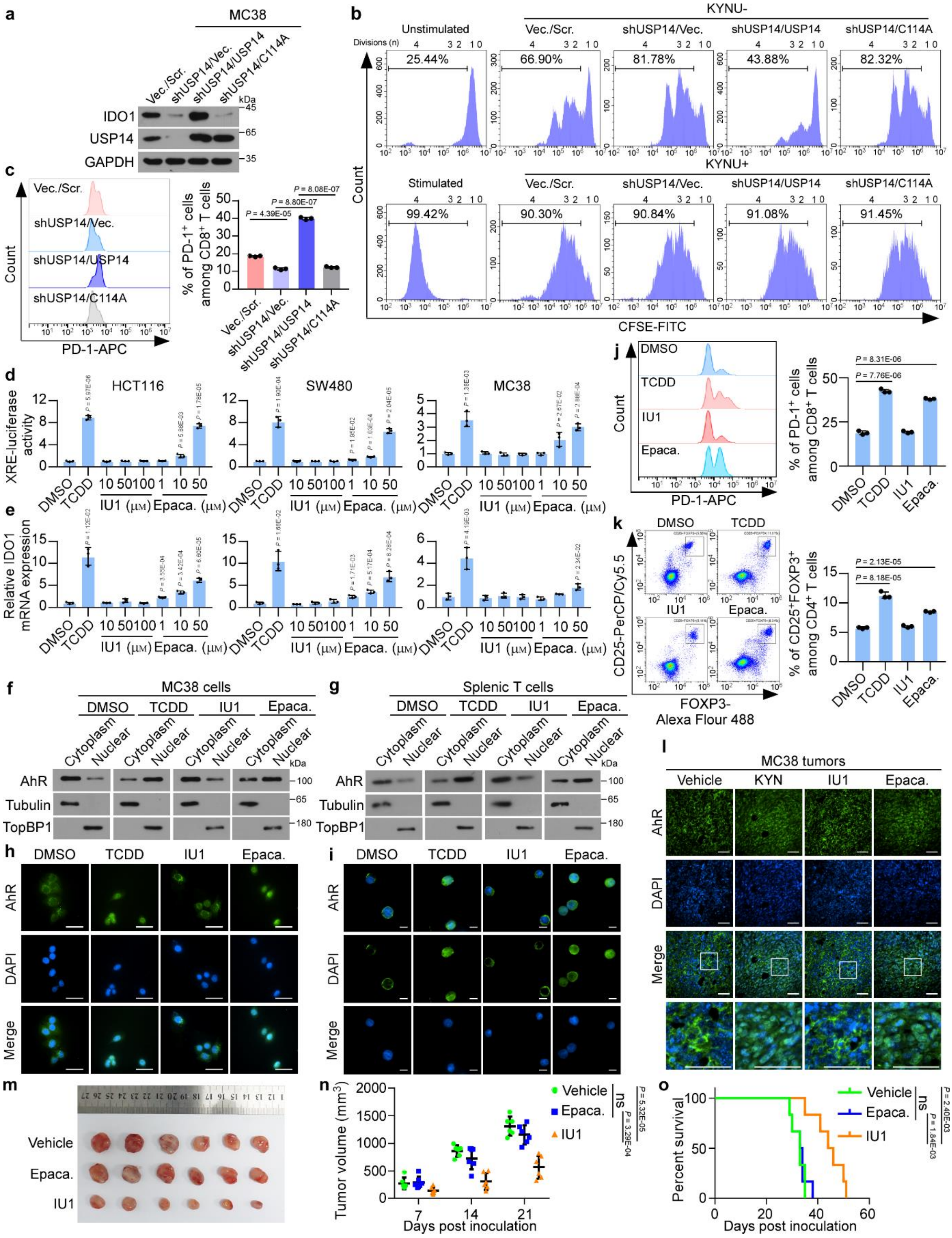

Supplementary Fig. 5. a Western blotting analysis of USP14 and IDO1 in MC38 cells reconstituted with *usp14* shRNA, *usp14* shRNA and *usp14*, or *usp14* shRNA and *usp14 C114A*. **b** Representative CFSE flow cytometry histograms of the proliferation of splenic CD8$^+$ T cells (n = 3 biological

replicates). **c** The percentage of PD-1^+ cells in $CD8^+$ T cells was measured by flow cytometry and analyzed by FlowJo software. **d** The indicated cells were treated with DMSO, TCDD (10 μM), IU1, or Epacadostat for 48 h. XRE-luciferase activity was measured and Firefly/Renilla luciferase ratio was normalized to DMSO. **e** The relative *IDO1* mRNA expression in the indicated cells treated with DMSO, TCDD (10 μM), IU1, or Epacadostat for 48 h. **f**, **g** MC38 cells (**f**) or splenic $CD8^+$ T cells (**g**) treated with DMSO, TCDD (10 μM), IU1 (50 μM), or Epacadostat (100 μM) for 48 h were fractionated and the cytoplasmic and nuclear protein fractions were analyzed with AhR, Tubulin (cytoplasmic marker), or TopBP1 (nuclear marker). **h**, **i** Representative images of AhR analyzed in MC38 cells (**h**) or splenic $CD8^+$ T cells (**i**) were treated with DMSO, TCDD (10 μM), IU1 (50 μM), or Epacadostat (100 μM) for 48 h. Scale bars, 50 μm (**h**), 10 μm (**i**). **j**, **k** Splenic $CD8^+$ T cells (**j**) or $CD4^+$ T cells (**k**) were treated with DMSO, TCDD (10 μM), IU1 (50 μM), or Epacadostat (100 μM) for 48 h. Percentage of PD-$1^+$$CD8^+$ T cells or $CD25^+$$FOXP3^+$ T cells was determined by flow cytometry and analyzed by FlowJo software. **l** Representative images of AhR in the indicated tumors. White squares indicated the represent image. Scale bars, 50 μm. **m**, **n** Representative micrographs and tumor volumes of the indicated tumors. n = 6 for each group. **o** Survival of the mice treated with IU1 or Epacadostat. ns, not significant, log-rank test. n = 6 for each group. In **c-e**, **j** and **k**, error bars represent the mean ± SD of three independent experiments. ns not significant, two-sided Student's t-test. Source data are provided as a Source Data file.

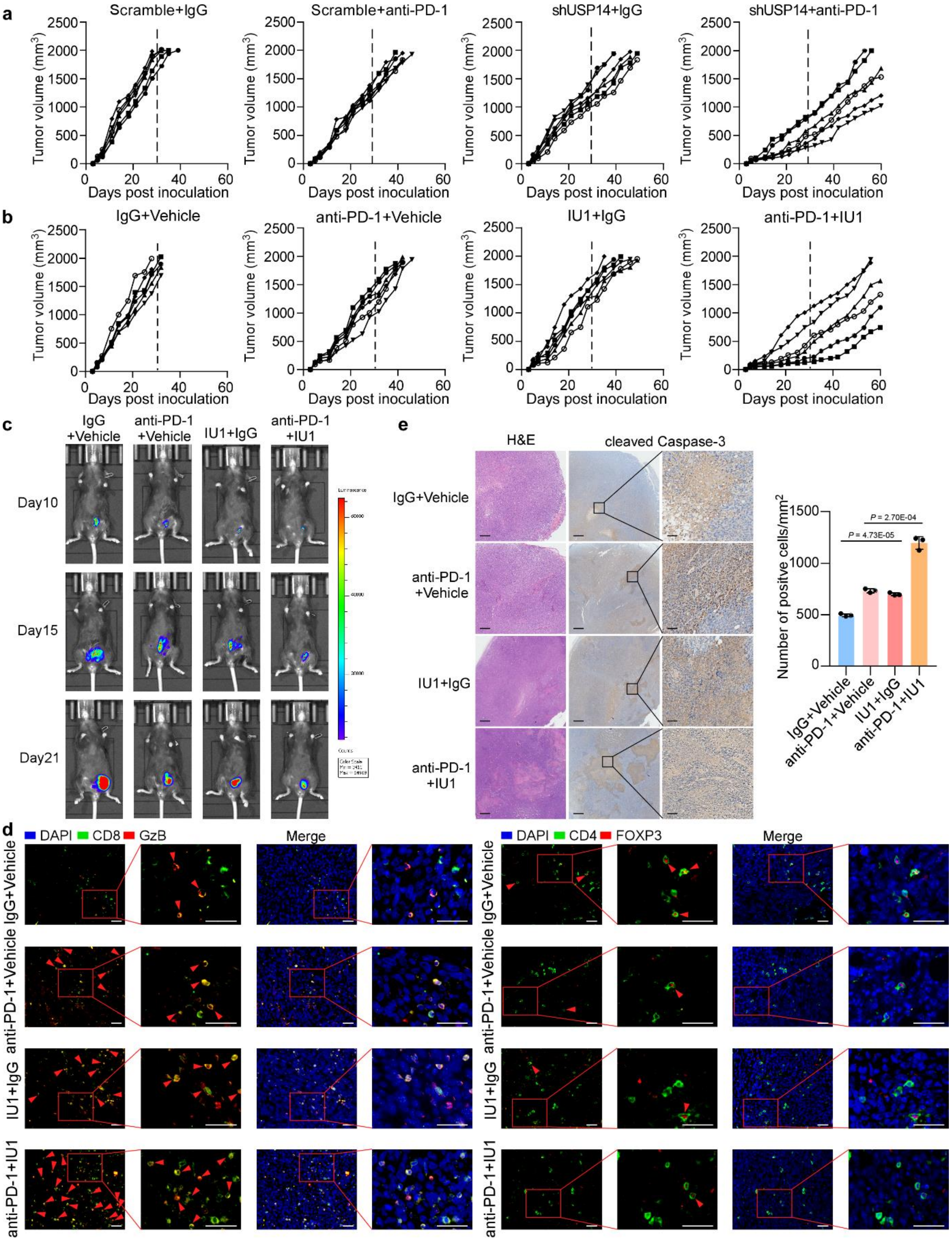

Supplementary Fig. 6. a Tumor growth rates were plotted for MC38-Scramble or MC38-shUSP14 tumors treated with anti-PD-1 or IgG isotype control. *n* = 6 for each group. **b** Tumor growth rates were plotted for MC38 tumors treated with IU1, anti-PD-1, or IU1 plus anti-PD-1. *n* = 6 for each group. **c**

Representative bioluminescence imaging of tumor-bearing mice at days 10, 15 and 21 post-tumor inoculation with IU1, anti-PD-1, or IU1 plus anti-PD-1. **d** Representative images of GzB^+CD8^+ cells and $FOXP3^+CD4^+$ cells analyzed by IF staining in tumors. The small red boxed areas were amplified images of cells. Multiple GzB^+CD8^+ cells and $FOXP3^+CD4^+$ cells can be observed in the tumors and were marked with red arrowheads. The far-right images in each panel were close-ups of the boxed region. GzB Granzyme B. Scale bars, 50 µm. **e** Representative images and quantification of cleaved caspase 3 (cCASP3) positive cells as analyzed by IHC staining. Scale bars, 500 µm (left panel), 100 µm (middle panel), 50 µm (right panel). Error bars represent the mean ± SD of three independent experiments, two-sided Student's *t*-test. Source data are provided as a Source Data file.

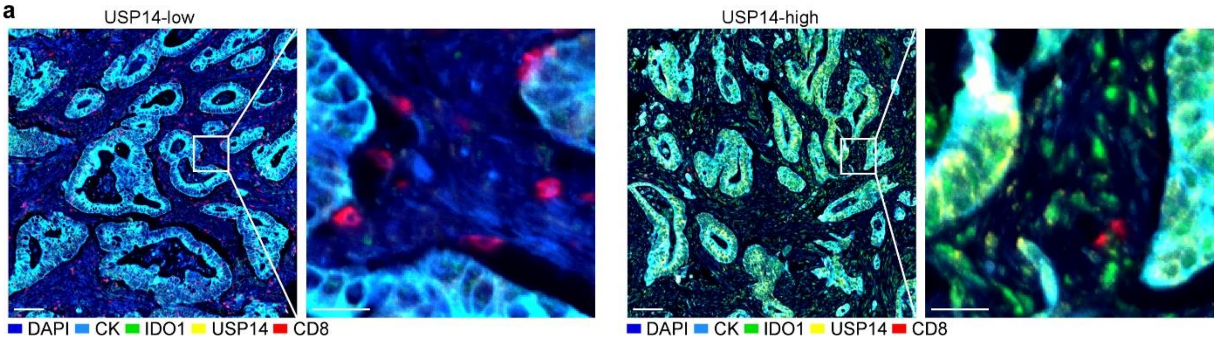

Supplementary Fig. 7. a Representative image showing multiplex immunofluorescence staining of two cases of CRC and CD8$^+$ TIL identification strategy. Each marker was represented by a different color as indicated in the panel, scale bars, 100 µm. White squares indicated the represent image, based in the CD8, IDO1, USP14 expression of the markers. The image shown in the white box is listed separately. CK cytokeratin. Scale bars, 25 µm. The experiment was repeated three times independently with similar results.

Supplementary Tables

Supplementary Table 1. Clinicopathological characteristics of 119 colorectal cancer patients.

Parameters	Number of cases (%)
**Age**	
< 55	60 (50.4)
≥ 55	59 (49.6)
**Clinical stage**	
I-II	84 (70.6)
III-IV	35 (29.4)
**T classification**	
T1-2	93 (78.2)
T3-4	26 (21.8)
**N classification**	
N0	40 (33.6)
N1-N3	79 (66.4)
**M classification**	
M0	104 (87.4)
M1	15 (12.6)
**Histologic grade**	
G1-2	94 (79.0)
G3	25 (21.0)
**Vital status**	
Alive	71 (59.7)
Dead	48 (40.3)
**IDO1 expression**	
Low	52 (43.7)
High	67 (56.3)
**USP14 expression**	
Low	42 (35.3)
High	77 (64.7)
**KYN expression**	
Low	83 (69.7)
High	36 (30.3)
**Immunoscore**	
Low	54 (45.4)
Intermediate	29 (24.4)
High	36 (30.2)

Supplementary Table 2. Correlation between USP14 and clinicopathological characteristics or IDO1 of colorectal cancer patients.

Characteristics	USP14 expression		*P* values
	Low, no. cases	High, no. cases	
**Age**			
< 55	22	38	0.752
≥ 55	20	39	
**Clinical stage**			
I-II	14	21	0.488
III-IV	28	56	
**T classification**			
T1-2	12	14	0.190
T3-4	30	63	
**N classification**			
N0	19	21	0.047
N1-3	23	56	
**M classification**			
M0	38	66	0.455
M1	4	11	
**Histologic grade**			
G1-2	32	62	0.580
G3	10	15	
**IDO1**			
Low	36	16	< 0.001
High	6	61	

Supplementary Table 3. Univariate and multivariate analysis of factors associated with 5-year overall survival in patients with CRC.

Characteristics	Univariate analysis		Multivariate analysis	
	HR (95% CI)	*P* values	HR (95% CI)	*P* values
**Age** (≥ 55 vs. < 55)	0.965 (0.548-1.700)	0.903	0.794 (0.433-1.457)	0.452
**Expression of USP14** (high vs. low)	3.317 (1.550-7.094)	0.002	3.182 (1.428-7.090)	0.005
**T classification** (T3-4 vs. T1-2)	5.403 (1.678-17.397)	0.005	2.232 (0.628-7.926)	0.214
**N classification** (N1-3 vs. N0)	4.906 (2.082-11.561)	< 0.001	3.164 (1.029-9.728)	0.044
**M classification** (M1 vs. M0)	4.364 (2.283-8.340)	< 0.001	3.674 (1.839-7.341)	< 0.001
**Histologic grade** (G3 vs. G1-2)	1.280 (0.746-2.758)	0.280	1.683 (0.841-3.365)	0.141
**Clinical stage** (III-IV vs. I-II)	3.634 (1.543-8.558)	0.003	1.055 (0.342-3.254)	0.925

HR, hazard ratio; CI, confidence interval.

Supplementary Table 4. The lists of primers used for qRT-PCR.

Primers		
**Primers for qRT-PCR**		**Sequence (5'-3')**
IDO1 (human)	Forward	GCCTGATCTCATAGAGTCTGGC
	Reverse	TGCATCCCAGAACTAGACGTGC
ido1 (mouse)	Forward	GCAGACTGTGTCCTGGCAAACT
	Reverse	AGAGACGAGGAAGAAGCCCTTG
GAPDH (human)	Forward	GTCTCCTCTGACTTCAACAGCG
	Reverse	ACCACCCTGTTGCTGTAGCCAA
gapdh (mouse)	Forward	CATCACTGCCACCCAGAAGACTG
	Reverse	ATGCCAGTGAGCTTCCCGTTCAG
**Target sequences**		**Sequence (5'-3')**
pSuper-retro-neo-*USP14*-shRNA#1 (human)		CCCAAGATTCAGCAGTCAGAT
pSuper-retro-neo-*USP14*-shRNA#2 (human)		GCAGCCAAATACAAGTGACAA
pSuper-retro-neo-*usp14*-shRNA#1 (mouse)		CCTTAGAAATTCTTGGCTGAA
pSuper-retro-neo-*usp14*-shRNA#2 (mouse)		TGCCTTATCTAACCAATATTT
pSuper-retro-neo-*ido1*-shRNA#1 (mouse)		CCTCGCAATAGTAGATACTTA
pSuper-retro-neo-*ido1*-shRNA#2 (mouse)		CGTCTCTCTATTGGTGGAAAT
pSuper-retro-neo-*TRIM21*-shRNA#1 (human)		TGAGAAGTTGGAAGTGGAAAT
pSuper-retro-neo-*TRIM21*-shRNA#2 (human)		TGGCATGGTCTCCTTCTACAA

Supplementary Table 5. List of used antibodies.

Antibodies	Catalogue No.	Company	Concentration	Application
Mouse Monoclonal anti-IDO1	#66528-1-Ig	Proteintech Group	1:1000	Western blotting
Rabbit Monoclonal anti-USP14	#11931	Cell Signaling Technology	1:1000	Western blotting
Rabbit Monoclonal anti-TRIM21	#92043	Cell Signaling Technology	1:1000	Western blotting
Rabbit Monoclonal anti-GAPDH	#5174	Cell Signaling Technology	1:5000	Western blotting
Rabbit Polyclonal anti-HA	#51064-2-AP	Proteintech Group	1:2000	Western blotting
Mouse Monoclonal anti-MYC	#60003-2-Ig	Proteintech Group	1:1000	Western blotting
Rabbit Monoclonal anti-Flag	#14793	Cell Signaling Technology	1:5000	Western blotting
Rabbit Polyclonal anti-ubiquitin	#3933	Cell Signaling Technology	1:1000	Western blotting
Mouse Monoclonal anti-Aryl hydrocarbon Receptor	#ab2769	Abcam	1:2000	Western blotting
Rabbit Monoclonal anti-tubulin	#5666	Cell Signaling Technology	1:1000	Western blotting
Rabbit anti-TopBP1	#PLA0030	Sigma-Aldrich	1:2000	Western blotting
Goat anti-rabbit immunoglobulin G	#ab7090	Abcam	1:2000	Western blotting
Goat anti-mouse immunoglobulin G	#ab97040	Abcam	1:2000	Western blotting
Rabbit Polyclonal anti-IDO1	#HPA023072	Sigma-Aldrich	1:500	Immunohistochemistry
Rabbit Polyclonal anti-USP14	#PA5-30300	Invitrogen	1:1000	Immunohistochemistry
Mouse Monoclonal anti-Kynurenine	#IS003	ImmuSmol	1:250	Immunohistochemistry
Rabbit Polyclonal anti-Cleaved Caspase-3	#9661	Cell Signaling Technology	1:400	Immunohistochemistry
Rabbit anti-CD3	#ZA-0503	ZSGB-BIO		Immunohistochemistry
Rabbit anti-CD8	#ZA-0508	ZSGB-BIO		Immunohistochemistry
Mouse Monoclonal anti-Granzyme B	#MA1-80734	Invitrogen	1:100	Immunofluorescence

Rat Monoclonal anti-CD8a	#42-0081-82	Invitrogen	1:200	Immunofluorescence
Rat Monoclonal anti-CD4	#92599	Cell Signaling Technology	1:200	Immunofluorescence
Rabbit Monoclonal anti-FOXP3	#12653	Cell Signaling Technology	1:200	Immunofluorescence
Rabbit Monoclonal anti-IDO1	#86630	Cell Signaling Technology	1:200	Immunofluorescence
Mouse Monoclonal anti-USP14	#67746-1-Ig	Proteintech Group	1:200	Immunofluorescence
Mouse Monoclonal anti-Kynurenine	#IS003	ImmuSmol	1:250	Immunofluorescence
Alexa Fluor 488 labeled anti-Rabbit	#A11008	Life Technologies	1:500	Immunofluorescence
Alexa Fluor 594 labeled anti-Rat	#A11007	Life Technologies	1:500	Immunofluorescence
Alexa Fluor 488 labeled anti-Mouse	#A11001	Life Technologies	1:500	Immunofluorescence
Alexa Fluor 594 labeled anti-Mouse	#A21203	Life Technologies	1:500	Immunofluorescence
Rabbit Monoclonal anti-CD3	#85061	Cell Signaling Technology	1:800	Multiple immunofluorescence
Mouse Monoclonal anti-CD8	#70306	Cell Signaling Technology	1:800	Multiple immunofluorescence
Rabbit Monoclonal anti-CD4	#48274	Cell Signaling Technology	1:800	Multiple immunofluorescence
Rabbit Polyclonal anti-IDO1	#HPA023072	Sigma-Aldrich	1:1000	Multiple immunofluorescence
Rabbit Polyclonal anti-USP14	#PA5-30300	Invitrogen	1:2000	Multiple immunofluorescence
Mouse Monoclonal anti-Keratin	#4545	Cell Signaling Technology	1:400	Multiple immunofluorescence
InVivo Monoclonal anti-Mouse PD-1 (clone RMP1-14)	#BE0146	BioXcell		In vivo
InVivo Monoclonal Rat IgG2a isotype control (clone 2A3)	#BE0089	BioXcell		In vivo
Rabbit Monoclonal anti-IDO1	#86630	Cell Signaling Technology		Immunoprecipitation
Rabbit Monoclonal anti-USP14	#11931	Cell Signaling Technology		Immunoprecipitation
Rabbit Monoclonal anti-TRIM21	#92043	Cell Signaling Technology		Immunoprecipitation

Supplementary Table 6. The antibody panels for sorting T cells by flow cytometry.

	Channel	Panel	Catalogue No.	Company
1	APC/Cyanine 7	CD45	#103116	Biolegend
2	Alexa Flour 488	FOXP3	#126405	Biolegend
3	PE/Cyanine 7	CD4	#100422	Biolegend
4	FITC	CD3e	#100203	Biolegend
5	PE	CD8a	#100707	Biolegend
6	PerCP/Cyanine 5.5	CD25	#101911	Biolegend
7	BV605	CD11b	#101237	Biolegend
8	APC	Granzyme B	#372203	Biolegend
9	GV405/BV421	Dead cells Dye	#13-0863-T100	Tonbo
10	APC	PD-1	#135209	Biolegend
