# Supplementary material for: USP14 promotes tryptophan metabolism and immune suppression by stabilizing IDO1 in colorectal cancer
Source: Nat Commun. 2022 Sep 26;13:5644. doi: 10.1038/s41467-022-33285-x (PMC9513055; doi:10.1038/s41467-022-33285-x)
Supplement: Supplementary file 3 — Description of Additional Supplementary Files [file 41467_2022_33285_MOESM3_ESM.docx]

**Description of Additional Supplementary Files**

**Supplementary Data 1.** The Mass Spectrometry results of HCT116 and SW480 cells.

**Supplementary Data 2.** The functional enrichment analysis results produced using gProfiler. The functional enrichment was analyzed by g:SCS method, which is the default method for computing multiple testing with correction for *P* - values gained from GO and pathway enrichment analyses.

**Supplementary Data 3.** Kynurenine and tryptophan levels in the supernatant of MC38-Vec./Scr., MC38-USP14/Scr., and MC38-USP14/shIDO1 cells according to ELISA. Two-sided Student’s t test was used to analyze the results.

**Supplementary Data 4.** Summary of TRP metabolism-associated metabolite levels in the plasma of mice with MC38-Vec./Scr., MC38-USP14/Scr., and MC38-USP14/shIDO1 tumors. Two-sided Student’s t test was used to analyze the results.

**Supplementary Data 5.** Kynurenine and tryptophan levels in the supernatant of MC38-Vec./Scr., MC38-shUSP14/Vec., MC38-shUSP14/USP14, and MC38-shUSP14/C114A cells according to ELISA. Two-sided Student’s t test was used to analyze the results.

**Supplementary Data 6.** Summary of TRP metabolism-associated metabolites levels in the plasma of mice with MC38-Vec./Scr., MC38-shUSP14/Vec., MC38-shUSP14/USP14, and MC38-shUSP14/C114A tumors. Two-sided Student’s t test was used to analyze the results.

**Supplementary Data 7.** Standard curve of the metabolites of TRP metabolism. Univariate linear regression analysis was used for each component.
